# Imbalance of the Immune Response According to Alcohol Consumption Patterns

**DOI:** 10.1155/mi/1693583

**Published:** 2025-10-16

**Authors:** Moises Martinez-Castillo, Abigail Hernandez-Barragan, Daniel Santana-Vargas, Zaira Medina-Avila, Marisela Hernandez-Santillan, Adrian Flores-Sanchez, Itzel Altamirano-Mendoza, Fatima Higuera-De La Tijera, Aldo Torre-Delgadillo, Jaqueline Cordova-Gallardo, José Luis Pérez-Hernández, Gabriela Gutierrez-Reyes

**Affiliations:** ^1^Liver, Pancreas and Motility Laboratory (HIPAM), Unit of Experimental Medicine, School of Medicine, Universidad Nacional Autónoma de México (UNAM), Mexico City, Mexico; ^2^Department of Gastroenterology, General Hospital of Mexico “Dr. Eduardo Liceaga”, Mexico City, Mexico; ^3^Research Department, General Hospital of Mexico “Dr. Eduardo Liceaga”, Mexico City 06720, Mexico; ^4^National Institute of Medical Sciences and Nutrition “Salvador Zubirán”, Mexico City, Mexico; ^5^General Hospital Dr. Manuel Gea González, Mexico City, Mexico

**Keywords:** alcohol-associated liver disease, alcohol consumption patterns, alcohol use disorders, cytokines, inflammation

## Abstract

**Background:**

Alcohol intake promotes the translocation of endotoxins, stimulating immune cell activation and the production of cellular mediators, dysregulating the inflammatory process. We simultaneously evaluated the number of immune cells and cytokine concentrations, in relation to the pattern of alcohol consumption.

**Methods:**

A cross-sectional study included five groups according to alcohol intake (hazardous drinking [HD], low alcohol use disorders [AUDs] [l-AUDs], moderate-severe AUDs [ms-AUDs], no decompensated cirrhosis, and alcohol-associated hepatitis [AH]). The control (CT) group was comprised of blood bank donors with an AUD Identification Test (AUDIT) <8 and occasional alcohol consumption of ≤10 g/day. Hematological and biochemical analyses were performed. Lymphocyte subsets (CD3^+^, CD8^+^, CD4^+^, natural killer [NK^+^], and NKT^+^cells) were determined using FACS analyses, whereas cytokine levels were determined using multiplex array technology. Multiple comparisons, Spearman correlations, calculated ratios, and receiver operating characteristic (ROC) curves were carried out.

**Results:**

A total of 780 subjects were enrolled and 427 were classified according to the alcohol consumption criteria. The ms-AUD group showed the highest levels of CD8^+^, NK, and NKT cells, whereas patients with cirrhosis had the lowest number of CD8^+^ subsets. AH displayed the highest number of neutrophils and cytokines. Moreover, lower levels of NK and NKT cells and upregulation of IL-6, CXCL-8, and TNF-α concentrations were observed, in accordance with alcohol intake (AH >cirrhosis > ms-AUD > l-AUD > HD).

**Conclusion:**

The number of immune cells (CD4^+^, CD8^+^, NK, and NKT cells) and IL-6, CXCL-8, IL-10, and TNF-α cytokine concentrations can be used as differential diagnostic parameters for AUD and could be considered an important criterion for the treatment of alcohol-associated liver disease (AALD).

## 1. Introduction

Alcohol is the most accepted addictive substance worldwide, and its consumption is associated with multiple health, economic, and social problems [1]. Alcohol consumption social protocols vary among cultures, with some showing greater acceptance [2]. Nevertheless, it has been estimated that approximately 75 million people worldwide present with alcohol use disorders (AUDs), conferring a high risk for developing alcohol-associated liver disease (AALD) [3]. AUD can induce cirrhosis and hepatocellular carcinoma, making it the main cause of liver damage. Cirrhosis and alcoholic hepatitis (AH) have negative social and health impacts; they affect the economy and are a burden to the health sector [4, 5].

According to the World Health Organization (WHO), moderate-severe AUD (ms-AUD) is defined as alcohol consumption ≥ 70 g/day in men and ≥ 50 g/day in women over the last 5 years [6]. However, alcohol consumption in these quantities is not considered a “serious” social or health problem [7]. Alcohol intake is a complex matter, because it involves biological, genetic, cultural, social, and psychological factors [2]. Furthermore, the specific criteria for defining the standard concentration of alcohol in drinks are unclear. Moreover, “normal” or “excessive” alcohol consumption usually involves personal ambiguity and social stigmas [2, 8–10]. The AUD Identification Test (AUDIT) and the Diagnostic and Statistical Manual of Mental Disorders (DSM) criteria are screening tools for detecting drinking patterns. Together, these tests make it possible to divide the classification into HD, l-AUD, and ms-AUD [10–13]. It is important to highlight that they do not take biochemical and clinical evidence into account, making them inaccurate and leading to a delay in the diagnosis of alcoholism or AUD, promoting progression to AALD or MetALD.

Initial alcohol metabolism begins in the duodenum, facilitating ulceration of the mucosa and erosions of the epithelium [14]. Moreover, alcohol metabolites, including acetaldehyde, can induce DNA adducts that cause cell injury [15]. Taken together, these pathological changes in the epithelium alter intestinal permeability and dysregulate the microbiota, promoting the translocation of endotoxin (LPS), via the porta, potentially inducing liver inflammation and fibrosis due to the activation of Kupffer cells and hepatic stellate cells [16, 17]. Both endotoxin and alcohol metabolism play pivotal roles in oxidative stress and inflammation of the liver parenchyma. The effects of alcohol on the immune response remain controversial. Some studies have shown that alcohol can interfere with the correct functioning of immune responses, and there is clinical evidence supporting the association between alcohol abuse and susceptibility to infectious diseases [18, 19]. However, until now, specific changes in immune function have not been fully elucidated and are usually considered clinically insignificant [18].

Immune cell regulation and cytokine production under different patterns of alcohol consumption have not been fully assessed. Currently, there is no biological/biochemical or clinical data available to diagnose alcohol-related health problems. These immunological mediators and biological molecules could be novel tools in frontier research for the prevention, diagnosis, and treatment of alcohol problems in AUD in the absence of clinical evidence and in AALD.

## 2. Materials and Methods

### 2.1. Subjects and Patients

This cross-sectional study enrolled a total of 1000 subjects recruited between 2013 and 2023. A total of 427 were classified according to alcohol consumption criteria, using the AUDIT test and DSM-IV and V, considering frequency, quantity, quality of alcohol, and clinical data. Nonetheless, the classification was adjusted with the DSM-V according to the number of positive answers, as mentioned below. Hazardous drinking (HD) was defined as an AUDIT > 8 and negative DSM-IV, whereas l-AUD was considered with an AUDIT > 8 and DSM-IV with 2–3 positive answers; alcohol dependance or ms-AUD was considered with an AUDIT > 8 and DSM-IV with more than 4–5 positive answers for moderate and >6 for severe. In addition, the ms-AUD classification was considered in accordance with the WHO alcoholism classification (alcohol consumption ≥ 70 g/day in men and ≥ 50 g/day in women over the last 5 years). The diagnoses of liver cirrhosis and AH included clinical and biochemical evidence. Imaging studies (ultrasound) were also performed to confirm the presence of advanced fibrosis with portal hypertension (portal vein diameter greater than 12 mm). Child–Pugh and MELD scores were used to classify the patients. Patients with cirrhosis, who presented with ascites, encephalopathy, or variceal bleeding at any point, were considered to have decompensated cirrhosis and were excluded from the study. For AH, the criteria of anemia, jaundice, thrombocytopenia, ascites, telangiectasias, hepatic insufficiency, and the Maddrey score were considered. The control (CT) group consisted of 353 blood bank donors from the Hospital General de México, with an AUDIT < 8 and occasional alcohol consumption of ≤ 40 g/occasion. Exclusion criteria for all groups were a positive serology for HIV and hepatitis A, B, and C, systemic infections (e.g., bacteria, flu, autoimmune diseases, etc.), and comorbidities (e.g., diabetes and hypertension), as well as patients with other concomitant liver damage, a history of traumatic brain injury, or central nervous system disorders. The procedure was approved by the institutional review boards of the Institutional Ethics Committees of the Hospital General de México (HG/DI/16/107/03/082) and the Universidad Nacional Autónoma de México (FMD/DI/15/2015). All participants provided written informed consent, and the study was conducted in accordance with the provisions of the Declaration of Helsinki.

### 2.2. Cohort Comparisons

Statistical analyses of demographic data included pairwise comparisons between CT groups and alcohol patterns: HD, l-AUD, ms-AUD, and in patients with AALD: cirrhosis, and AH by age. Random CT participants were selected from a database and compared with each pattern of alcohol consumption and AALD (compensated cirrhosis and AH).

### 2.3. Clinical and Biochemical Data

The evaluation procedures included a detailed physical examination and anthropometric information, such as sex, age, body mass index (BMI) (kilograms/meters^2^; weight/height^2^), and nutritional status. Alcohol consumption was evaluated using the AUDIT, DSM-IV, and DSM-V, frequency, and grams of alcohol per occasion and per day. Biochemical parameters included bilirubin (total and direct), albumin, glucose, aspartate aminotransferase (AST), alanine aminotransferase (ALT), and gamma glutamyl transpeptidase (GGT). Hematological data included total leukocytes, lymphocytes, monocytes, neutrophils, eosinophils, and basophils, which were analyzed and compared. Biochemical and hematological tests were performed with automated systems (Vitros 250, Johnson and Johnson, New Jersey, USA and HMX-AL Hematology Analyzer Beckman Coulter California, USA).

### 2.4. Evaluation of the Lymphocyte Subpopulations

Blood samples from each subject were collected by venipuncture; the sample was maintained with EDTA and then processed for flow cytometric analyses. A total of 50 µL of blood was incubated with a mixture of monoclonal antibodies to define discrete T cell subsets. The commercial antibody mixture contained antibodies against the following lymphocyte subsets: total T cells (CD3^+^), T-helper cells (CD3^+^ and CD4^+^), T-cytotoxic cells (CD3^+^ and CD8^+^), natural killer (NK) cells (CD3-, CD16^+^, and CD56^+^), and NKT cells (CD3^+^, CD16^+^, and CD56^+^) (Becton Dickinson, San Jose, CA). Samples were treated with 1 × BD Pharm Lyse and washed with sterile PBS 1 ×. After adjusting for forward scatter (FSC) and side scatter (SSC), 50,000 events were quantified per sample, and the data were analyzed on a CANTO II flow cytometer using BD FACS Diva software (V. 6.1.3). The results were expressed as the percentage of positive cells.

### 2.5. Determination of Cytokine Profiles

Ten milliliters of blood were used to evaluate cytokine concentration. The blood samples were centrifuged at 3500 rpm/10 min, and the serum was recovered and stored at −80°C, until use. The simultaneous determination of IL-2, IL-4, IL-6, CXCL-8, IL-10, and TNF-α was performed using multiplex suspension array technology (Millipore, Billerica, MA, USA). Nontreated serum samples from patients and CTs (25 µL) were evaluated using the HCYTOMAG-60K kit, with no cross-reactivity and minimal intra- and interassay errors (%CV <10) (Merck, Millipore, USA). The data were acquired using Luminex200 MAGPIX Systems, following the supplier's specifications (series number 10294005; Merck, Millipore, USA). The data were validated with internal standards and CTs; cytokine concentrations were obtained using Luminex XPONENT software and compared with the minimum and maximum detection values for each cytokine.

### 2.6. Statistical Analysis

A pairwise comparison of CT versus other groups was made, considering age range and sex as parameters. For this, CT datasets were randomly selected using the “random sample of cases” function of the SPSS “Data→ Select Cases” menu. CT datasets were selected, according to three times the number of each alcohol intake group, except when the number of subjects was insufficient, such as in the cirrhosis group, which had 1.5-times the number of CT versus cirrhotic patients. Qualitative variables were expressed as counts and percentages and expressed as means and standard errors. Pairwise comparisons were performed using the Mann–Whitney *U* test, and the minimum (min), maximum (max), and quartiles (Q1–Q3) were determined. The cytokines analyzed included IL-2, IL-4, IL-6, CXCL-8, IL-10, and TNF-α. The principal component analysis (PCA) was used to reduce dimensionality and identify major patterns in serum cytokine profiles across study groups. Prior to the PCA, cytokine concentrations were transformed using the log(1 + x) function to minimize the impact of extreme values and to address skewed distributions due to lower detection limits, especially for IL-2 and TNF-α. After log-transformation, data were standardized using *Z*-score normalization (mean-centered and scaled to unit variance). The PCA was performed using the scikit-learn library (version 1.4.1) in Python 3.11. The first two principal components were retained for visualization and interpretation, and a biplot was generated to display both the distribution of samples and the contribution of each cytokine. Confidence ellipses (95%) were constructed for each group to visualize the dispersion and overlap among conditions. Bivariate Spearman correlations were used to compare biochemical parameters between the alcohol intake groups. The ratios of pro- and anti-inflammatory cytokines were then calculated. Lymphocyte subpopulations and cytokines from alcohol consumption groups were assessed with the receiver operating characteristic (ROC) curves and area under the ROC curve (AUC), and the cutoff values were identified with the Youden's index. Differences were considered statistically significant when the *p*-value was less than 0.05. Effect sizes were calculated from the standardized Mann–Whitney *U* test and the square root of the total observations and interpreted according to using effect size was calculated using Cohen's *r* as part of the statistical strategy. This measure provided an estimate of the magnitude of the association between variables, complementing *p*-values by indicating the practical relevance of the findings. Values of *r* were interpreted according to Cohen's benchmarks (0.10 = small, 0.30 = medium, and 0.50 = large). The statistical analysis was performed using IBM SPSS Statistics for Windows, Version 22 (IMB Corp., Armonk, NY). Cytometry and cytokine data were plotted using GraphPad Prism Software V6 (La Jolla, CA, USA). The statistical analyses used in this study were performed by a biomedical statistician.

## 3. Results

### 3.1. Distribution of Patients and CT Subjects

A total of 780 participants fulfilled the inclusion criteria. The CT group included 353 subjects, made up of 144 women (age range: 18–56 years) and 209 men (age range: 18–61 years) (Supporting Information 1: Figure S1). In contrast, 427 patients were selected according to alcohol consumption patterns (HD = 80, I-AUD = 42, ms-AUD = 122, cirrhosis = 121, and AH = 62). Age adjustment showed statistical differences between the cirrhosis group and the CT subjects due to the intrinsic condition of chronic disease and the donor criteria employed (*p*  < 0.001) (Supporting Information 1: Figure S1). The above mentioned group distribution was used to evaluate the subsequent data analysis.

**Figure 1:**
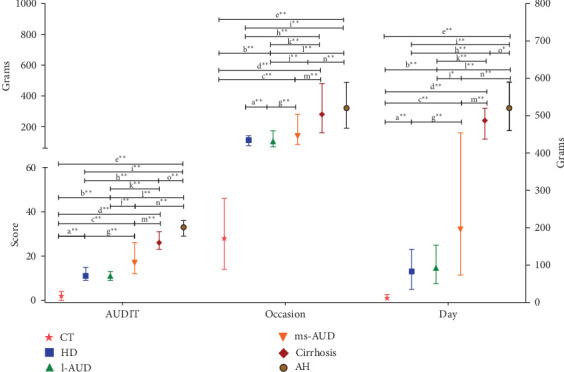
AUDIT and alcohol consumption according to alcohol patterns. The AUDIT score (0–40 points) of each alcohol consumption, as well as the grams of alcohol per occasion and per day, were analyzed in each alcohol pattern. Data were analyzed by the Mann–Whitney *U* test and expressed as median and standard error. a, Control (CT) versus hazardous drinking (HD); b, CT versus low alcohol use disorders (l-AUDs); c, CT versus moderate and severe alcohol use disorders (ms-AUDs); d, CT versus cirrhosis; e, CT versus AH; f, HD versus l-AUD; g, HD versus ms-AUD; h, HD versus cirrhosis; i, HD versus AH; j, l-AUD versus ms-AUD; k, l-AUD versus cirrhosis; l, l-AUD versus AH; m, ms-AUD versus cirrhosis; n, ms-AUD versus AH; o, cirrhosis versus AH. *⁣*^*∗*^*p*  < 0.01, significant; *⁣*^*∗∗*^*p*  < 0.001, very significant.

For the distribution group, we performed a comparison of alcohol intake using alcohol consumed per occasion and per day. No significant differences were observed in the CT subgroups (data not shown); thus, their combined average was used in graphs for comparisons (Figure 1). The AUDIT and alcohol consumption revealed a gradually increased score (Figure 1). In the AH group, the AUDIT revealed an increased score, with a maximum of 33 points. Alcohol intake in grams per occasion and per day displayed a continuous elevation in alcohol patterns, mainly in ms-AUD, cirrhosis, and AH, and statistical differences were also calculated in all groups studied (Figure 1).

### 3.2. Biochemical Changes in the Different Patterns of Alcohol use

After confirming the correct alcohol pattern classification, we analyzed the BMI and biochemical parameters. The CT subgroups were classified as overweight according to BMI (Supporting Information 2: Table S1). The alcohol intragroup analysis revealed that l-AUD was within the normal BMI range and HD, ms-AUD, cirrhosis, and AH were overweight (Table 1).

To compare biochemical changes according to alcohol intake, we compared each group, and its respective CT (Supporting Information 1: Figure S1). Patients with cirrhosis and AH showed higher total bilirubin levels than those with HD, l-AUD, and ms-AUD (Table 1). The direct bilirubin concentrations were within the normal range in the cirrhosis group, whereas patients with AH had elevated direct bilirubin concentrations, reflecting liver decompensation (Table 1). Albumin levels decreased in chronic AALD and AH, which could be associated with reduced hepatic function. Furthermore, transaminases (AST, ALT, and GGT) showed an increase in a consumption-dependent manner in the cirrhosis and AH groups, achieving maximum values in AH (Table 1). Glucose levels were normal in all groups but were higher in patients with cirrhosis and AH (Table 1 and Supporting Information 2: Table S1).

Furthermore, the correlation analysis of biochemical parameters in the alcohol intake groups revealed that the AUDIT and GGT presented a moderately positive correlation in the HD group (0.336, *p*  < 0.01), whereas in ms-AUD, total bilirubin was positively correlated with AST (0.646, *p*  < 0.001), ALT (0.444, *p*  < 0.01), and GGT (0.391, *p*  < 0.05); direct bilirubin was positively correlated with AST (0.476, *p*  < 0.01) and GGT (0.380, *p*  < 0.05); and ms-AUD showed a negative correlation between albumin and AST (−0.556, *p*  < 0.001), direct bilirubin (−0.537, *p*  < 0.001), total bilirubin (−0.433, *p*  < 0.01), and GGT (−0.345, *p*  < 0.05). Contrastingly, in the cirrhosis group, AST was moderately correlated with total bilirubin and direct bilirubin levels (0.532, *p*  < 0.001; 0.579, *p*  < 0.001, respectively). A positive correlation was also observed between ALT and total bilirubin levels (0.396, *p*  < 0.001). In contrast, albumin levels were negatively correlated with total bilirubin (−0.507, *p*  < 0.001), direct bilirubin (−0.554, *p*  < 0.001), and AST (−0.457, *p*  < 0.001).

### 3.3. Changes in the Number of Peripheral Blood Cells by Alcohol Intake Groups

The populations of neutrophils, lymphocytes, monocytes, eosinophils, and basophils were evaluated in the CT subgroups (Supporting Information 3: Table S2-A, Table 1). The AH group displayed the highest number of leukocytes, which may be related to the systemic inflammatory response and infectious processes (Table 1). A large effect size was found in HD versus AH (*r* = 0.50; *p*  < 0.001), ms-AUD versus AH (*r* = 0.45; *p*  < 0.001), and cirrhosis versus AH (*r* = 0.48; *p*  < 0.001). Neutrophils showed a marked increase in the AH group when compared with the ms-AUD (*r* = 0.44; *p*  < 0.001) and cirrhosis (*r* = 0.55; *p*  < 0.001) groups. There were fewer lymphocytes in the cirrhosis group than the other groups (Table 1), whereas monocytes increased in alcoholic cirrhosis and AH (Table 1). The evaluation of eosinophils and basophils showed no statistical differences in any of the groups evaluated (data not shown). It is important to mention that even though there were no statistical differences in the total leukocyte number in the ms-AUD group, compared with the other consumption patterns, the results showed changes in the number of lymphocytes, monocytes, and neutrophils, in relation to their corresponding CT groups, as a possible result of the early effect of alcohol on these immune cells (Supporting Information 3: Table S2-A).

The correlation analysis of peripheral blood cells from the l-AUD group showed that lymphocytes correlated positively with monocytes (0.421, *p*  < 0.05). In the ms-AUD, there was a positive correlation between monocytes and grams of alcohol/occasion (0.397, *p*  < 0.01) and neutrophils (0.312, *p*  < 0.01), as well as a negative correlation between albumin and monocytes (−0.502, *p*  < 0.05) and leukocytes (−0.380, *p*  < 0.05). In cirrhosis, leukocytes were moderately correlated with direct bilirubin (0.440, *p*  < 0.001), GGT (0.391, *p*  < 0.001), and total bilirubin (0.388, *p*  < 0.001). Neutrophils positively correlated with total bilirubin and direct bilirubin (0.371, *p*  < 0.01; 0.43, *p*  < 0.001, respectively), as well as with GGT (0.361, *p*  < 0.01). Furthermore, monocytes were correlated with neutrophils (0.536, *p*  < 0.001) and lymphocytes (0.548, *p*  < 0.001), and neutrophils were correlated with lymphocytes (0.452, *p*  < 0.001). In AH, there was a significant correlation between leukocyte and neutrophil counts (0.630, *p*  < 0.05). In this group, there was also a high positive correlation between neutrophils and the AUDIT (0.746, *p*  < 0.01), and a moderately positive correlation between neutrophils and GGT (0.654, *p*  < 0.05). In addition, the monocytes in these patients correlated with alcohol consumption (grams of alcohol intake/day) (0.633, *p*  < 0.05) and grams of alcohol intake/occasion (0.633, *p*  < 0.05). Interestingly, the correlation of leukocytes and neutrophils was the only correlation that was shared in HD (0.712, *p*  < 0.001), l-AUD (0.899, *p*  < 0.001), ms-AUD (0.891, *p*  < 0.001), cirrhosis (0.892, *p*  < 0.001), and AH (0.63, *p*  < 0.01)

### 3.4. Alteration of CD4^+^, CD8^+^, NK, and NKT Cells, in Accordance With Alcohol Intake Patterns

**Figure 2:**
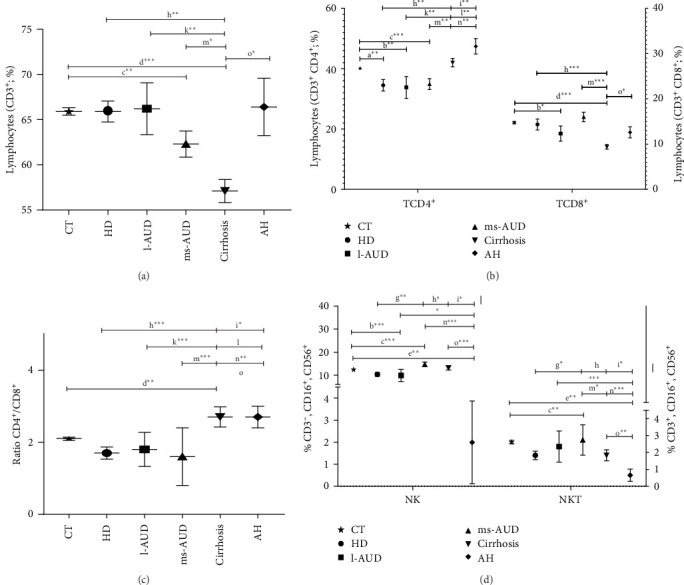
Comparison of CD4^+^, CD8^+^, NK, and NKT cells in alcohol consumption patterns. (a) Comparison of CD3^+^ lymphocytes in hazardous drinking (HD), low alcohol use disorders (l-AUDs), moderate and severe alcohol use disorders (ms-AUDs), and cirrhosis and alcoholic hepatitis (AH). (b) Comparison of the CD4^+^ and CD8^+^ lymphocyte subsets in HD, l-AUD, ms-AUD, cirrhosis, and AH. (c) CD4^+^/CD8^+^ ratio from each alcohol pattern studied. (d) Analysis of NK (CD3^−^, CD16^+^, and CD56^+^) and NKT (CD3^+^, CD16^+^, and CD56^+^) in each alcohol pattern. The data were obtained by FACS and expressed as percentage, data were analyzed by the Mann–Whitney *U* test and expressed as median, and standard error. a, Control (CT) versus hazardous drinking (HD); b, CT versus low alcohol use disorders (l-AUDs); c, CT versus moderate and severe alcohol use disorders (ms-AUDs); d, CT versus cirrhosis; e, CT versus AH; f, HD versus l-AUD; g, HD versus ms-AUD; h, HD versus cirrhosis; i, HD versus AH; j, l-AUD versus ms-AUD; k, l-AUD versus cirrhosis; l, l-AUD versus AH; m, ms-AUD versus cirrhosis; n, ms-AUD versus AH; o, cirrhosis versus AH. *⁣*^*∗*^*p*  < 0.05, significant; *⁣*^*∗∗*^*p*  < 0.01, very significant; *⁣*^*∗∗∗*^*p*  < 0.001, highly significant.

Lymphocyte subpopulations included CD4^+^, CD8^+^, NK, and NKT cells. Before evaluating each lymphocyte subpopulation, the parameters of each cell lineage were adjusted (Supporting Information 4: Figure S2). No differences were observed in any of the CT subgroups (Supporting Information 3: Table S2 -B). Because no significant differences were observed among the CT subgroups (Supporting Information 3: Table S2-B), their combined average was used as the reference CT for comparisons with the various alcohol consumption patterns. The HD group showed a reduction in CD4^+^ lymphocytes, compared with the CT group (*r* = 0.37; *p*  < 0.01) (Figure 2b), as a possible result of the early effect of alcohol on the immunological response. Differences in the CD4^+^ (*r* = 0.18; *p*  < 0.01) and CD8^+^ (*r* = 0.12; *p*  < 0.05) counts were observed between the l-AUD and CT groups (Figure 2b). Similarly, ms-AUD displayed fewer lymphocytes (CD3+, *r* = 0.22; *p*  < 0.01) and CD4^+^ lymphocyte subsets (*r* = 0.28; *p*  < 0.001), compared with the CT group (Figure 2a, b). Lymphopenia (CD3^+^, *r* = 0.44; *p*  < 0.01) was evident in cirrhosis versus CT, and CD8^+^ (*r* = 0.53; *p*  < 0.01), and the CD4^+^/CD8^+^ ratio (*r* = 0.37; *p*  < 0.01) versus CT (*p*  < 0.001) (Figure 2). In contrast, there was a significant increase in CD4^+^ cells in the CT group, compared with the HD (*r* = 0.37; *p*  < 0.01), l-AUD (*r* = 0.18; *p*  < 0.01), and ms-AUD groups (*r* = 0.28; *p*  < 0.01) (Figure 2b), whereas the CD8^+^ subset had the lowest number in the cirrhosis group versus the same groups HD (*r* = 0.10; *p*  < 0.01), l-AUD (*r* = 0.12; *p*  < 0.01), and ms-AUD groups (*r* = 0.05; *p*  < 0.01). When the CD4^+^/CD8^+^ ratio was analyzed, the data showed a significant increase in the CD4^+^/CD8^+^ ratio in patients with cirrhosis versus HD (*r* = 0.95; *p*  < 0.001), l-AUD (*r* = 0.35; *p*  < 0.001), and ms-AUD (*r* = 0.56; *p*  < 0.001), which could be related to the advanced stage of fibrosis (Figure 2c). AH had the highest percentage of CD4^+^, compared with the l-AUD (CD4^+^*r* = 0.18; *p*  < 0.01) and ms-AUD (CD4^+^*r* = 0.18; *p*  < 0.01) groups (Figure 2a,b). Nevertheless, there was a significant increase in the percentage of CD8^+^ lymphocytes in AH, compared with cirrhosis (*r* = 0.15; *p*  < 0.05), possibly because of the acute effect of alcohol (Figure 2b). Additionally, the CD4^+^/CD8^+^ ratio was significantly higher in AH, compared with the HD (*r* = 0.16; *p*  < 0.05) and ms-AUD (*r* = 0.14; *p*  < 0.01) groups (Figure 2c).

Regarding NK cells, ms-AUD had higher levels than the CT group, as well as the highest levels of NKT cells (*r* = 0.23; *p*  < 0.001 and *r* = 0.21; *p*  < 0.01, respectively) (Figure 2d). Cirrhosis also presented an increase in the proportion of NK cells, compared with the CT group (*r* = 0.28; *p*  < 0.001), HD (*r* = 0.45; *p*  < 0.05), and AH (*r* = 0.25; *p*  < 0.001) (Figure 2b). In contrast, a decrease in the level of NKT cells was found in cirrhosis, compared with ms-AUD (*r* = 0.26; *p*  < 0.05) (Figure 2d). AH displayed an evident decrease in NK cells and NKT cells, compared with all the groups evaluated (alcohol intake and CT groups), with moderate Cohen's values for NK in ms-AUD (*r* = 0.20; *p*  < 0.001) and cirrhosis (*r* = 0.25; *p*  < 0.001) comparisons (Figure 2d). In summary, lymphocyte CD3^+^ levels were regulated as follows: AH = l-AUD >HD >ms-AUD >cirrhosis. The increment in CD4^+^ was AH >cirrhosis >ms-AUD >l-AUD >HD, and in CD8^+^, was ms-AUD >HD >AH = l-AUD >cirrhosis. The CD4^+^/CD8^+^ ratio was AH = cirrhosis >l-AUD >HD >ms-AUD; NK cells were ms-AUD >cirrhosis >l-AUD >AH; and NKT cells were ms-AUD >l-AUD >HD = cirrhosis >AH.

### 3.5. Dysregulation of Cytokine Production During Alcohol Consumption

**Figure 3:**
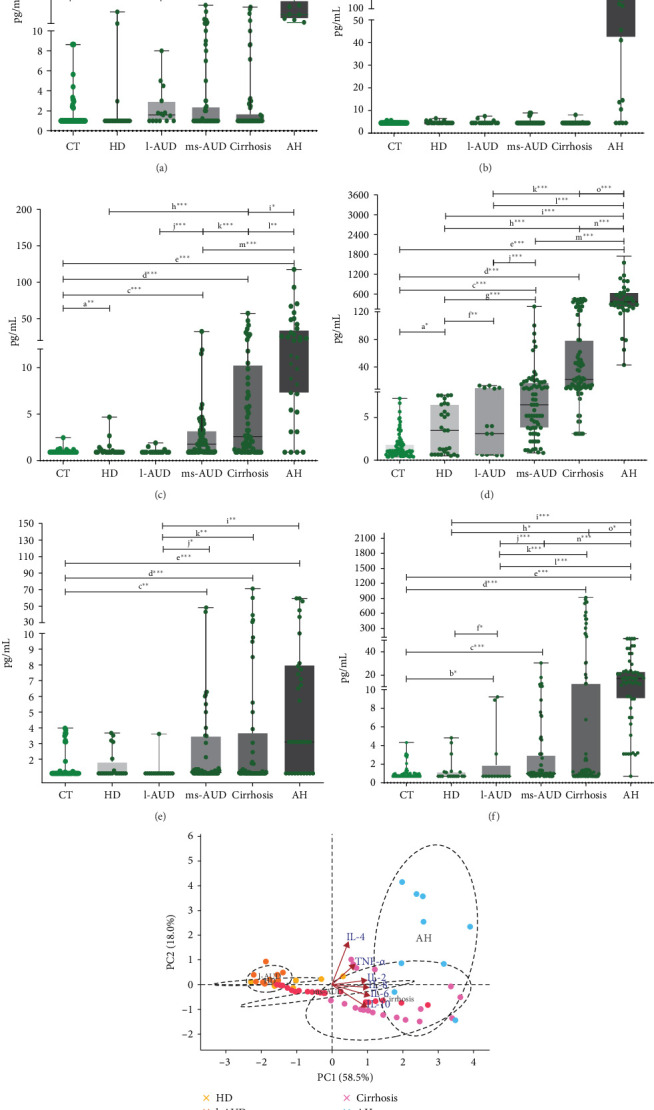
Serum cytokine concentration and biplot of PCA in the alcohol consumption patterns. (a) Serum concentration of IL-2 in the subjects with HD, l-AUD, ms-AUD, cirrhosis, and AH. (b) Serum concentration of IL-4, (c) IL-6, (d) CXCL-8, (e) IL-10, and (f) TNF-α in the alcohol intake groups and their respective CT groups. Multiplex suspension array technology was used and pairwise concentration data were analyzed using the Mann–Whitney *U* test and expressed as median, min, and max (bars). (g) Biplot shows the distribution of individual samples based on the first two principal components (PC1 and PC2), which together explain 76.5% of the total variance. Each point represents a sample, colored by study group, and ellipses represent the 95% confidence region for each group. Arrows indicate the direction and relative contribution of each cytokine to the components. a, Control (CT) versus hazardous drinking (HD); b, CT versus low alcohol use disorders (l-AUDs); c, CT versus moderate and severe alcohol use disorders (ms-AUDs); d, CT versus cirrhosis; e, CT versus AH; f, HD versus l-AUD; g, HD versus ms-AUD; h, HD versus cirrhosis; i, HD versus AH; j, l-AUD versus ms-AUD; k, l-AUD versus cirrhosis; l, l-AUD versus AH; m, ms-AUD versus cirrhosis; n, ms-AUD versus AH; o, cirrhosis versus AH. *⁣*^*∗*^*p*  < 0.05, significant; *⁣*^*∗∗*^*p*  < 0.01, very significant; *⁣*^*∗∗∗*^*p*  < 0.001, highly significant.

**Figure 4:**
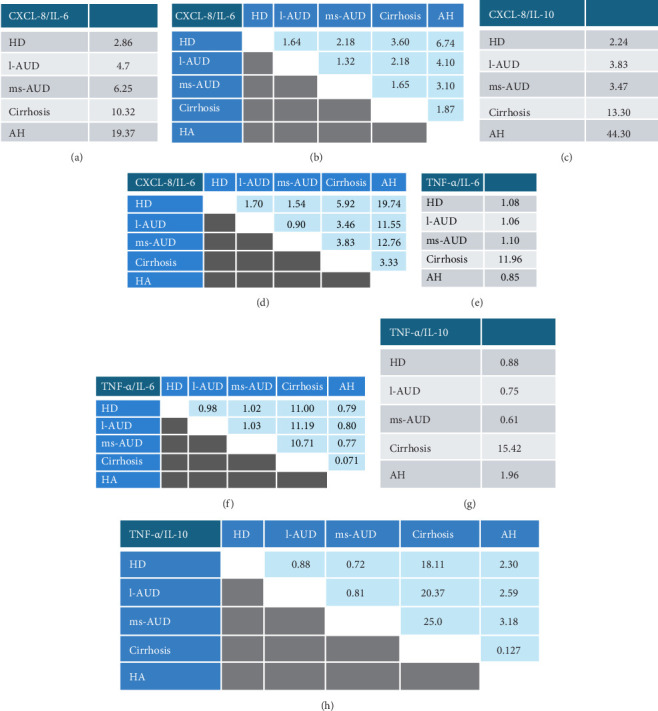
Calculated ratio of inflammatory and anti-inflammatory cytokines. Ratio estimation of (a) CXCL-8/IL-6, (c) CXCL-8/IL-6, (e) TNF-α/IL-6, and (g) TNF-α/IL-10, in each alcohol consumption pattern. Comparative ratio analysis between alcohol consumption patterns; (b) CXCL-8/IL-6, (d) CXCL-8/IL-6, (f) TNF-α/IL-6, and (h) TNF-α/IL-10. Hazardous drinking (HD), low alcohol use disorders (l-AUDs), moderate and severe alcohol use disorders (ms-AUDs) and alcoholic hepatitis (AH). Statistical means were used to calculate the ratio.

After evaluating the regulation of different cell lineages, a profile of cytokines, including IL-2, IL-4, IL-6, CXCL-8 (also termed CXCL-8/IL-8), IL-10, and TNF-α, was determined. The statistical analysis of each cytokine in the CT subgroups showed no differences, as expected (data not shown). However, we observed differences in IL-4, IL-6, and CXCL-8 levels in the HD group, compared with the combined mean of the CT subgroups (Figure 3). Similarly, alcohol intake in the l-AUD group revealed upregulation of IL-2, CXCL-8, and TNF-α versus its CT subgroup (Figure 3, Supporting Information 5: Table S3). The ms-AUD, cirrhosis, and AH groups showed significant differences in all evaluated cytokines, as well as in the clinical relevance calculated by the size effect (Cohen's values were determined) (Supporting Information 6: Table S4). The concentrations of IL-2, IL-4, IL-6, CXCL-8, and TNF-α were higher in the ms-AUD group than in the l-AUD group (Figures 3a-d and f). Intragroup differences between cirrhosis and ms-AUD revealed higher concentrations of IL-6 (*r* = 0.30; *p*  < 0.001) and CXCL-8 (*r* = 0.42; *p*  < 0.001) (Figures 3c,d). Serum concentrations of IL-2, IL-4, CXCL-8, and TNF-α were higher in AH than in cirrhosis (*p*  < 0.001) (Figures 3a,b,d,f Supporting Information 5: Table S3). The highest concentration of IL-10 was observed in AH. Cirrhosis and ms-AUD displayed a significant increase in IL-10 levels, compared with the CT groups (*r* = 0.27; *p*  < 0.01, *r* = 0.78; *p*  < 0.001, respectively) (Figure 3, Supporting Information 5: Table S3, Supporting Information 6: Table S4). IL-6, CXCL-8/IL-8, and TNF-α levels increased according to alcohol consumption pattern and liver damage: ms-AUD, cirrhosis, and AH (Figure 3, Supporting Information 5: Table S3, Supporting Information 6: Table S4). The highest concentrations of cytokines in the AH group were an invariable result of the intense inflammatory response and infection process in this clinical condition. Moreover, the PCA showed that PC1 and PC2 explained 76.5% (95% confidence interval [CI]) of the total variance in cytokine concentrations, with PC1 accounting for 58.5% and PC2 for 18.0% (95% CI) (Figure 3g). PC1 was predominantly associated with proinflammatory cytokines, including IL-6, CXCL-8, IL-10, and IL-2, indicating that this component captured an overall inflammatory response gradient. In contrast, PC2 was largely influenced by IL-4 and TNF-α, suggesting an axis of variation related to immune regulation or Th2-like responses. The direction and length of cytokine vectors in the biplot reflected their relative contribution to the variability captured by each component (Figure 3g). To explore the orchestration of inflammatory and anti-inflammatory responses, we considered IL-6 as having an anti-inflammatory role, resulting in the CXCL-8/IL-6, CXCL-8/IL-10, TNF-α/IL-6, and TNF-α/IL-10 ratios. We found that the CXCL-8/IL-6 ratio showed a progressive increase in accordance with the AUD status (Figure 4a). Importantly, in that ratio, CXCL-8 increased more than two times in HD, close to five times in l-AUD, and approximately six times in ms-AUD; in cirrhotic patients, the difference was 10 times, and in AH it was 20 times (Figure 4a). Moreover, the calculated ratio increased in accordance with the differences between the consumption patterns and AALD. This means that the comparison of distant patterns (AH vs HD) showed greater differences in the calculated ratio (CXCL8/IL-6 = 6.74) (Figure 4b); in contrast, the comparison of closer patterns, such as l-AUD versus HD, showed fewer differences in the calculated ratio (CXCL8/IL-6 = 1.64) (Figure 4b). A similar performance was observed for CXCL8/IL-10, where CXCL-8 increased progressively in accordance with AUD (Figure 4c). The comparison between groups showed an evident increment in CXCL-8 over IL-10, when the reference group was AH (44 times), and the maximum value ratio was observed in AH versus HD (19.74 times of CXCL-8 over IL-10) (Figure 4d).

The TNF-α/IL-6 and TNF-α/IL-10 ratios showed that TNF-α was approximately 12 and 25 times more elevated than IL-6 and IL-10 in cirrhosis, respectively. However, the TNF-α/IL-6 and TNF-α/IL-10 ratios in HD, l-AUD, and ms-AUD showed that TNF-α only increased approximately one magnitude in relation to the cytokines analyzed (Figure 4e, g). Interestingly, in AH, the ratio also showed a modest twofold increase in TNF-α over IL-10. Equally, the contrast ratio showed great elevation of TNF-α, IL-6, and IL-10 in cirrhosis (Figure 4f, h).

### 3.6. Immune Cells and Cytokines as Discriminators in Alcohol Pattern Consumption

After analyzing the changes in biochemical parameters, proportion of peripheral blood cells, concentration of cytokines, correlation analysis, and calculated ratios, we performed ROC curves, determining the cutoff values, sensitivity (SE), and specificity (SP) of each parameter.

**Figure 5:**
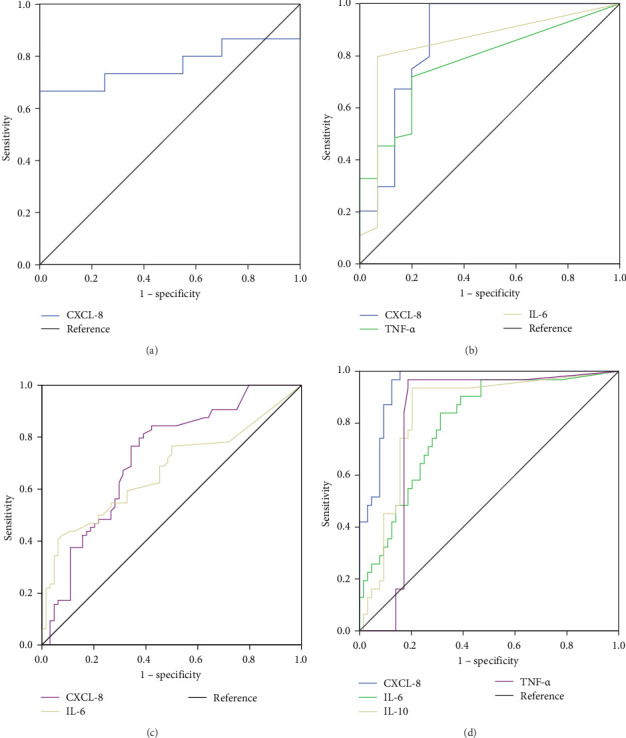
Cytokines as discriminators in alcohol pattern consumption. (a) The ROC curve of CXCL-8 showed an AUC of 0.76 (95% CI: 0.56–0.95; *p*  < 0.013) in HD versus l-AUD. (b) In l-AUD versus ms-AUD, IL-6, CXCL-8, and TNF-α showed an AUC of 0.77 (95% CI: 0.65–0.90; *p*  < 0.001), 0.86 (95% CI: 0.73–0.99; *p*  < 0.0001), and 0.85 (95% CI: 0.73–0.96; *p*  < 0.0001), respectively. (c) In ms-AUD versus cirrhosis, IL-6 and CXCL-8 displayed an AUC of 0.67 (95% CI: 0.57–0.76; *p*  < 0.001) and 0.72 (95% CI: 0.63–0.81; *p*  < 0.0001). (d) In cirrhosis versus AH, the IL-6, CXCL-8, IL-10, and TNF-α showed an AUC of 0.79 (95% CI: 0.70–0.88), 0.95 (95% CI: 0.91–0.99), 0.83 (95% CI: 0.75–0.93), and 0.81 (95% CI: 0.71–0.91), all of them with a *p*  < 0.0001.

**Figure 6:**
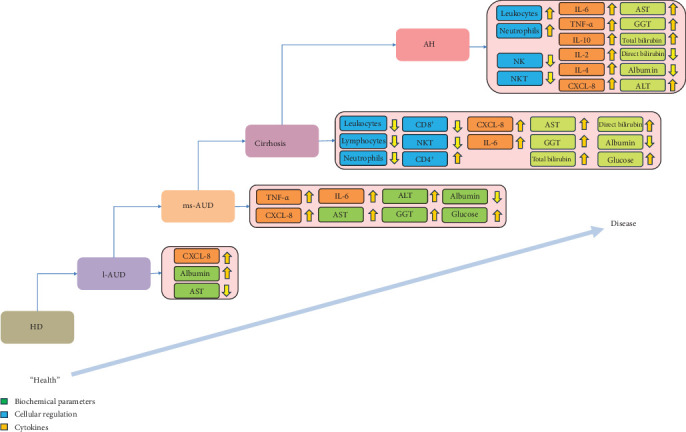
Biochemical and immune discriminators in accordance with the AUD and AALD. Representation of progressive immunological changes according to harmful alcohol consumption showed over- or downregulation of several biochemical, cellular, and immune mediators (cytokines). Illustrative images were performed using calculated ROC values. Hazardous drinking (HD), low alcohol use disorders (l-AUDs), moderate and severe alcohol use disorders (ms-AUDs), and alcoholic hepatitis (AH) were described. Statistical means were used to calculate the ratios. Note the biochemical values (dark green) with normal values.

In our statistical model we found that CXCL-8 was the only discriminating factor at early stages of alcohol consumption, showing an AUC of 0.76 (95% CI [0.56–0.95], *p*  < 0.013) (Figure 5a). The cutoff point for discriminating l-AUD from HD was 0.8 pg/mL, with 73.3 (95% CI; 48–89.1) SE and 70.6 (95% CI; 56.6–87.3) SP (Table 2). The other consumption comparisons showed the addition of other discriminators; for example, there were significant differences in l-AUD versus ms-AUD, IL-6, CXCL-8, and TNF-α (Figure 5b, Table 2). In a similar manner, in ms-AUD versus cirrhosis, CXCL-8 and IL-6 were the soluble discriminators (Figure 5c, Table 2). However, in cirrhosis versus AH, the soluble mediators, CXCL-8, IL-6, IL-10, and TNF-α, showed a good AUC (Figure 5d, Table 2). The other comparisons of consumption pattern versus HD showed the progressive addition of more discriminators. For example, in AH versus HD, the neutrophil, IL-6, CXL-8, IL-10, and TNF-α values were higher than 90 in the AUC, with a *p*  < 0.0001 and 95% CI in closed intervals of 0.87–0.99 (Table 2). Moreover, the important factors in relation to the comparative groups are summarized in Table 2, allowing discrimination of the level of change in accordance with alcohol intake. Nevertheless, the summarized analysis of severity in accordance with AUD and liver disease is: (HD🡪l-AUD🡪ms-AUD🡪cirrhosis🡪AH) and is visualized in Figure 6, where the AUC values were employed to discriminate the increase or decrease of the representative element or discriminator (immune cells and cytokines).

## 4. Discussion

Moderate alcohol consumption has been considered beneficial [20, 21]. However, recent controversial evidence strongly suggests that, under certain circumstances, the beneficial properties are doubtful, even at low concentrations [22–26]. In this context, several efforts have been made in past years to create a definition of a standard drink, because the recommended doses are usually not included on beverage labels [27–30]. Unfortunately, the public misinterprets or is not familiar with these considerations when they acquire and consume alcoholic beverages.

It is important to note that the DSM-V is currently used to evaluate alcohol consumption. However, in this study, we used both the DSM-IV and DSM-V because of the timing of the study population recruitment. Nevertheless, the criteria for AUD were unified into low, moderate, and severe subclassifications, without affecting the changes observed in the immune response reported herein [31, 32].

The early stages of AUD (HD and l-AUD) are commonly evaluated through conventional alcohol tests (AUDIT, DSM, and CAGE). In alcohol dependance or ms-AUD subjects, the most sensitive laboratory tests include GGT, mean corpuscular volume, and the ALT/AST ratio, but the AUDIT and CAGE questionnaires have been considered the best screening tools [33, 34]. It is important to highlight that the early stages of AUD are pivotal in developing strategies to prevent its progression. Unfortunately, without intervention, AUD progresses to AALD, given that there are no approved biomarkers for AUD. In this sense, our data showed no changes in the AUDIT scores, grams of alcohol consumed, or the frequency of alcohol consumption in the HD and l-AUD groups. In fact, they exhibited similar patterns across all the evaluated parameters. Thus, it is possible that several subjects in the HD group did not provide accurate information and may have belonged to the l-AUD group. This close relationship underscores the need to develop novel biological and biochemical markers, instead of relying on tools that depend on subjective self-reporting and memory.

On the other hand, in our study, the values of bilirubin, albumin, glucose, AST, ALT, and GGT showed overproduction mainly in the transition between l-AUD/ms-AUD, ms-AUD/cirrhosis, and in AH, correlating with previously reported data [35, 36]. Using the Spearman rank correlation, our study results showed that, in the ms-AUD group, total bilirubin was positively correlated with AST, ALT, and GGT, whereas albumin displayed a negative correlation. Similar results were observed in the cirrhosis group, as previously reported [37], suggesting that these parameters can be used as indicators of progressive liver damage in AALD.

In addition, the direct and indirect effects of acute and chronic alcohol consumption, resulting in hematological adverse events, have been previously reported [38–40]. Direct consequences include toxicity to the bone marrow, blood cell precursors, and erythrocytes. Alcohol abuse has also been reported to cause myelosuppression and to reduce all blood cell types [41, 42]. In 2017, changes in the number of white blood cells were reported according to alcohol consumption levels [42]. Our findings correlated with these changes, mainly in cirrhotic patients, where the total number of lymphocytes decreased, which has been associated with nutritional deficiencies [43]. In contrast, we observed an excessive number of total leukocytes and neutrophils in AH patients. This dysregulation can be produced by the formation of adducts due to acetaldehyde and ROS overproduction [14, 44]. The effect of alcohol on human neutrophils is not fully understood. However, the presence of polymorphonuclear neutrophils (PMNs) in the liver has been suggested to compromise hepatocyte survival [45]. We want to emphasize that our findings showed increased circulating neutrophils mainly in patients with AH, which is in line with recent studies demonstrating that AH, compared with alcohol-associated cirrhosis, is characterized by the expansion of a self-sustaining population of IL-8^+^ neutrophils [46]. In the same study, the authors performed single-cell RNA-Seq and immunostaining analyses, showing that up to 70% of liver nonparenchymal cells in AH were IL-8^+^ neutrophils, whereas this population is practically absent in cirrhosis [46].

Not only are these IL-8^+^ neutrophils responsive to IL-8, but they also produce IL-8 themselves, establishing a feed-forward loop that perpetuates neutrophil recruitment and activation. Mechanistically, IL-8^+^ neutrophils display high expressions of TNF-α and IL-1 receptors and activation of the p38 MAPK pathway, further amplifying inflammatory responses within the liver [46]. The presence of neutrophils in the systemic circulation may occur because of their recruitment to the liver or as an incursion of these cell lineages to eliminate adducts at the peripheral level. Moreover, ethanol promotes alterations in the gut-liver axis via microbial dysbiosis and loss of intestinal barrier integrity, in turn inducing a severe inflammatory response, which is a central characteristic in the progression of AH [47]. In fact, bacterial translocation has been considered an important pathogenic driver of systemic inflammation in acute-on-chronic liver failure [48]. During these events, both PAMPs (e.g., LPS) and DAMPs (e.g., mitochondrial DNA from cellular injury and death) can travel in the portal circulation, promoting intrahepatic neutrophil infiltration. Additionally, high intrahepatic neutrophils and low levels of CD8^+^ T cells are clearly distinguished subtypes of AH. Furthermore, patients with severe AH demonstrated a high systemic neutrophil-to-lymphocyte ratio, which has been shown to be predictive of poor clinical outcomes [47]. Importantly, pentoxifylline and prednisolone are used in the treatment of AH [49, 50]. Prednisolone is a glucocorticoid (GC) known to exert negative effects on T lymphocytes and to act as a nonspecific phosphodiesterase inhibitor, showing inhibition of TNF production in in vivo and in vitro studies [50, 51]. In addition, GCs are considered the standard of care for severe AH, but there is now contradictory evidence regarding the effect of GCs on neutrophil functions [52]. Under specific conditions, the use of GCs promotes anti-inflammatory or proinflammatory effects or can even promote or inhibit apoptosis in neutrophils [52]. Ronchetti et al. [52] suggested that these differential effects most likely depend on the underlying disease and the immunological microenvironment. Additionally, some patients with AH do not respond positively to prednisolone, whereas others are not candidates for treatment with GCs [52, 53]. Thus, targeting IL-8^+^ neutrophils and the chemokyne CXCL-8 represents a promising but untested therapeutic avenue in AH that merits further preclinical and clinical investigation, with careful attention to patient safety and infection risk.

Furthermore, a refined analysis of lymphocyte subpopulations showed the early impact of alcohol on CD4^+^, CD8^+^, NK, and NKT cells. Interestingly, alcohol promoted an increase in CD4^+^ cells in an alcohol-dependent manner (l-AUD <HD <ms-AUD <cirrhosis <AH), whereas for CD8^+^, it was cirrhosis <AH <ms-AUD. CD8^+^ T cells have been reported to have impaired cytotoxic functions and reduced activation in patients with AH, contributing to susceptibility to infection [54]. Moreover, we observed that ms-AUD displayed higher numbers of NK cells and NKT cells, but the biological implications and cell status need to be evaluated, including their activation and effector capacity [38, 39]. In chronic alcoholic liver disease (cirrhosis and AH), the reduction of peripheral NK cells and the suppression of their functions have been previously reported [39, 55]. Additionally, decreased activation and degranulation capacity in NK cells has been reported in patients with AH [54]. Herein, we provide valuable evidence that the CD4^+^/CD8^+^ ratio, NK cells, and NKT cells can be used as indicators of immunological alterations in AUD and AALD, which can be helpful in the clinical context.

Alcohol consumption alters immunological mediators, including cytokines, resulting in changes in the plasma, liver, lung, and brain levels [56, 57]. However, the role of IL-2 in chronic liver disease remains unclear. Lower serum activity of this cytokine in the chronic phase of AALD has been reported in in vitro studies [19, 58], but there is limited information on IL-2 levels in patients, with respect to alcohol consumption. Higher levels of IL-2 have been observed in cirrhotic patients, correlating with our findings [59]. Those authors reported that IL-2 plays a pivotal role in the activation of follicular T helper cells [59].

Similarly, we found elevated production of IL-4 in the serum of patients with AH. In contrast, in in vitro studies, treatment of B cells with ethanol (150 mg) resulted in decreased IL-4 production [60]. Changes in IL-2 and IL-4 have been suggested to be associated with recurrent gastrointestinal infection susceptibility [19]. Circulating concentrations of TNF-α, IL-1 α, IL-1β, IL-6, CXCL-8, IL-12, and monocyte chemoattractant protein-1 (MCP-1) have been considered promising candidates for diagnosing l-AUD [57, 61]. Our data on dysregulation of IL-6, CXCL-8, and TNF-α correlated with other studies on AALD [57, 61]. We also demonstrated an increase in the levels of these cytokines following harmful alcohol consumption and AALD.

Furthermore, in our analysis, PCA distribution and the separation of groups suggest that healthy CT tend to cluster toward lower PC1 scores, reflecting a low-inflammatory baseline, whereas clinical groups (e.g., l-AUD, ms-AUD, cirrhosis, and AH) are distributed along higher PC1 values, indicative of elevated systemic inflammation. This separation reinforces the biological relevance of the cytokine profiles and supports their use as potential biomarkers of immune status or disease severity. The evident discriminatory power of the calculated ratios reported herein may also be applied clinically as promising indicators of alcohol consumption severity, regardless of whether it is the cause or the consequence. Moreover, in AH patients, the following approaches may be considered: neutralization of CXCL-8 with specific antibodies (such as ABX-IL-8 or HuMax-IL-8/BMS-986253); antagonism of the CXCL-8 receptors CXCR1/2, for which pepducin-based or small-molecule inhibitors have shown efficacy in murine models of alcohol-related steatohepatitis; or inhibition of upstream signaling pathways that drive CXCL-8 production, such as p38 MAPK.

In recent years, the immunological outcomes of alcohol, including changes in immune mediator cells and circulating cytokines, have become clearer. At first glance, our data may appear to be an extensively descriptive analysis. However, it delves deeply into understanding the inflammatory milieu associated with alcohol consumption, extending from initial stages to the severe dysregulation observed in cirrhosis and AH. Thus, in the near future, the immune cells and cytokines analyzed may be used as complementary indicators to determine patient status. Moreover, this information needs to be used efficiently in the context of the development of novel therapeutic alternatives for the treatment and CT of AUD and AALD. Although we excluded comorbidities and controlled population conditions in our study, it is important to consider other factors that contribute to the production and activation of lymphocytes and cytokines, including nutrition and drug use. Nevertheless, this study on a Latin American (Mexican) population is a strong reinforcement in the field of alcohol consumption and immunological changes at the clinical level, from the early stages of hazardous alcohol intake.

It is important to emphasize that harmful alcohol consumption causes approximately 6% of total deaths worldwide due to cirrhosis. In addition, severe AH has an elevated mortality rate, ranging from 28 days to 6 months, even with the use of prednisolone. Unfortunately, there are no standardized definitions of different drinking patterns due to the lack of an accurate and validated tool or diagnostic method to differentiate them. Moderate or abusive alcohol consumption is usually a subjective assessment. Currently, no biological or clinical data are available for the diagnosis of alcohol-related health problems.

## 5. Conclusion

The results of our study provide strong evidence that some biochemical parameters and several immune response mediators are directly related to alcohol consumption patterns, starting with hazardous alcohol consumption. Thus, the number of immune cells (CD4^+^, CD8^+^, NK^+^, and NKT^+^) and serum cytokine levels (IL-6, CXCL-8, and TNF-α) can be used as biochemical tools for the diagnosis of AUD and as important criteria for the treatment of ms-AUD. Strong immune dysregulation is observed in liver cirrhosis, but the overproduction of cytokines, resembling a cytokine storm, occurs in AH. Thus, immune mediators can be used as biomarkers and as new targets for therapies in AALD.

## 6. Limitations of the Study

The main limitation of this study is the absence of longitudinal data, which hinders the understanding of how immune and cytokine profiles evolve over time, with changing alcohol consumption patterns. For instance, it is not possible to determine whether the immune dysregulation detected in hazardous drinkers would normalize with abstinence or worsen with continued drinking. However, the study enables the identification of correlations between immune cells and cytokines based on alcohol consumption, providing a foundation for alternative clinical therapeutic interventions. Moreover, another important limitation is the lack of objective biomarkers, such as phosphatidylethanol, to avoid misclassification potentially caused by subjective tests (AUDIT and DSM). While this novel strategy is primarily used in studies on treatment adherence, the alcohol concentration estimated in the present study through the personalized questionnaire revealed clear differences, supporting the accuracy of the classification. Finally, further mechanistic exploration is required to determine whether these immune response changes drive ALD progression or are a consequence of alcohol intake.

## Figures and Tables

**Table 1:** Biochemical and hematological parameters in the different alcohol pattern groups.

Parameter	HD (*n* = 80)	l-AUD (*n* = 42)	ms-AUD (*n* = 122)	Cirrhosis (*n* = 121)	AH (*n* = 62)	*p*-Value
BMI (Kg/m^2^)	26.17 ± 0.4 (23–28.3)	24.6 ± 0.5 (22.1–26.9)	25.49 ± 0.36 (23.3–28.7)	27.6 ± 0.45 (24–30.5)	27 ± 0.7 (23.6–29.9)	b^⁣^*∗*^^, h^⁣^*∗*^^, j^⁣^*∗*^^, l^ȣ^, m^⁣^*∗*^^

Total bilirubin (mg/dL)	0.78 ± 0.05 (0.5–1.0)	0.7 ± 0.05 (0.5–0.8)	1.1 ± 0.1 (0.6–1)	3.9 ± 0.5 (1.2–4.6)	20.1 ± 1.4 (14.5–27)	c*⁣*^*∗*^, d^§^, e^§^, h^§^, i^§^, k^§^, l^§^, m^§^, n^§^, o^§^

Direct bilirubin (mg/dL)	0.16 ± 0.02 (0.1–0.2)	0.1 ± 0.04 (0.1–0.1)	0.2 ± 0.08 (0.1–0.2)	1.85 ± 0.36 (0.3–1.9)	11.6 ± 0.8 (7.7–14.7)	a^*⁣*^*∗*^^, c^§^, d^§^, e^§^, h^§^, i^§^, k^§^, l^§^, m^§^, n^§^, o^§^

Albumin (mg/dL)	4.3 ± 0.1 (1. 4–4.7)	4.7 ± 0.09 (4.5–4.9)	4.0 ± 0.02 (3.9–4.5)	2.9 ± 0.07 (2.2–3.5)	2.5 ± 0.35 (1.7–2.4)	b^ȣ^, c^§^, d^§^, e^§^, g^*⁣*^*∗*^^, h^§^, i^§^, j^§^, k^ȣ^, l^ȣ^, m^§^, n^§^, o^§^

Glucose (mg/dL)	94 ± 3.2 (84–98)	90 ± 4.7 (74–108.3)	93.8 ± 2.29 (84–100)	105.3 ± 3.8 (88–107)	103.4 ± 3.1 (88.8–119)	e*⁣*^*∗*^, h^ȣ^, i^*⁣*^*∗*^^ k^ȣ^,,l^*⁣*^*∗*^^,m^*⁣*^*∗*^^,n^*⁣*^*∗*^^

AST (IU/L)	38 ± 4.1 (22–37)	30 ± 3 (20–34)	34.5 ± 1.7 (24–40)	69.5 ± 5.22 (38–75)	165.7 ± 10.1 (114–188)	a^*⁣*^*∗*^^, c^§^, d^§^, e^§^, h^§^, i^§^, j^*⁣*^*∗*^^, k^§^, l^§^, m^§^, n^§^, o^§^

ALT (IU/L)	35.4 ± 3.8 (18–36)	32.3 ± 6.9 (16–32)	32.8 ± 2.1 (20–38)	41.75 ± 2.8 (24–47)	53.15 ± 3.22 (34–67)	c^§^, d^§^, e^§^, h^ȣ^, i^§^, j^*⁣*^*∗*^^, k^§^, l^§^, m*⁣*^*∗*^, n^§^, o^§^

GGT (IU/L)	55.8 ± 13.3 (19–45)	25.3 ± 1.94 (16–30)	50 ± 6.2 (19–49)	158.5 ± 18.2 (48–192)	372 ± 44.9 (111.8–531)	a*⁣*^*∗*^, c^§^, d^§^, e^§^, h^§^, i^§^, j^ȣ^, k^§^, l^§^, m^§^, n^§^, o^§^

Leukocytes (10^3^/mm^3^)	6.8 ± 0.25 (5.5–7.9)	7.1 ± 0.4 (5.3–8.4)	6.7 ± 0.22 (5.8–7.8)	7.2 ± 0.4 (4.4–9)	16.1 ± 1.2 (11–20.4)	e^§^, i^§^, l^§^, n^§^, o^§^

Lymphocytes (10^3^/mm^3^)	2 ± 0.14 (1.5–2.5)	2 ± 0.1 (1.6–2.3)	2.0 ± 0.07 (1.7–2.3)	1.46 ± 0.08 (1–2)	3.1 ± 0.8 (1–4.9)	a^*⁣*^*∗*^^, b^*⁣*^*∗*^^, h^ȣ^, k^ȣ^, m^§^

Monocytes (10^3^/mm^3^)	0.4 ± 0.4 (0.28–0.5)	0.4 ± 0.03 (0.2–0.6)	0.4 5 ± 0.02 (0.3–0.5)	0.5 ± 0.05 (0.4–0.7)	0.8 ± 0.1 (0.5–1.2)	e^ȣ^, h*⁣*^*∗*^, i^ȣ^, k*⁣*^*∗*^, l^ȣ^, n^ȣ^, o*⁣*^*∗*^

Neutrophils (10^3^/mm^3^)	3.9 ± 0.2 (3.1–4.8)	4.3 ± 0.3 (3.2–5.6)	4.1 ± 0.1 (3.3–5)	4.4 ± 0.4 (2.2–5.7)	13.1 ± 1.8 (6.9–19.2)	c^*⁣*^*∗*^^, e^§^, i^§^, l^§^, m*⁣*^*∗*^, n^§^, o^§^

*Note:* a, Control (CT) versus hazardous drinking (HD); b, CT versus low alcohol use disorders (l-AUDs); c, CT versus moderate and severe alcohol use disorders (ms-AUDs); d, CT versus cirrhosis; e, CT versus AH; f, HD versus l-AUD; g, HD versus ms-AUD; h, HD versus cirrhosis; i, HD versus AH; j, l-AUD versus ms-AUD; k, l-AUD versus cirrhosis; l, l-AUD versus AH; m, ms-AUD versus cirrhosis; n, ms-AUD versus AH; o, cirrhosis versus AH. Data were analyzed using the Mann–Whitney *U* test and expressed as mean ± EE and interquartile ranges (Q1–Q3).

Abbreviations: ALT, alanine aminotransferase; AST, aspartate aminotransferase; GGT, gamma-glutamyl transferase.

*⁣* ^*∗*^ *p*  < 0.05, Significant.

^ȣ^ *p*  < 0.01, Very significant.

^§^ *p*  < 0.001, Highly significant.

**Table 2:** AUC of discriminators or parameters and alcohol consumption patterns.

Comparative groups	Parameter	Directive	AUC (95% CI)	*p*-Value	Cutoff	SE (95% CI)	SP (95% CI)
HD versus l-AUD	CXCL-8	—	0.76 (0.56–0.95)	0.013	0.8	73.3 (48.0–89.1)	70.6 (56.6–87.3)

HD versus ms-AUD	NK	—	0.67 (0.58–0.76)	0.001	12.1	64.8 (52.9–80.5)	58.9 (29.2–76.6)
	IL-6	—	0.71 (0.58–0.83)	0.008	0.93	71.9 (50.4–78.4)	64.7 (42.0–87.4)
	CXCL-8	—	0.77 (0.63–0.90)	0.001	3.6	75 (68.3–91.7)	70.6 (48.9–92.3)
	TNF-α	—	0.66 (0.50–0.83)	0.037	0.72	79.7 (68.3–91.7)	64.7 (42.0–87.4)

HD versus cirrhosis	Monocytes	—	0.66 (0.54–0.78)	0.013	0.43	59.1 (47.2–71.0)	67.9 (50.6–85.2)
	NK	—	0.62 (0.53–0.72)	0.016	10.05	62.7 (53.2–70.3)	50 (49.5–70.4)
	CD8+	Negative	0.72 (0.63–0.82)	0.0001	17.45	73.3 (61.4–81.2)	71.4 (57.3–80.5)
	CD4+	—	0.66 (0.57–0.76)	0.001	39.05	65.3 (54.6–75.5)	64.3 (51.9–76.0)
	IL-6	—	0.81 (0.72–0.90)	0.0001	1.07	75 (63.2–84)	76.5 (53.1–88.8)
	CXCL-8	—	0.90 (0.82–0.98)	0.0001	4.16	90.6 (84.0–96.4)	76.5 (8.1–85.5)
	TNF-α	—	0.65 (0.52–0.80)	0.049	0.71	64.1 (58.3–78.4)	64.7 (38.6–79.7)

HD versus AH	Monocytes	—	0.78 (0.60–0.96)	0.006	0.45	81.8 (55.2–95.3	67.9 (49.3–82.1)
	Neutrophils	—	0.93 (0.85–1)	0.0001	4.85	81.8 (60.1–96.0)	75 (56.6–87.3)
	NK	Negative	0.71 (0.53–0.90)	0.019	4.1	66.7 (35.9–91.8)	78.6 (66.4–92.7)
	NKT	Negative	0.77 (0.62–0.91)	0.004	0.87	83.3 (52.9–97.8)	73.2 (66.4–92.7)
	CD4+	—	0.78 (0.63, 0.93)	0.003	44.65	66.7 (35.5–82.3)	85.7 (74.7–92.7)
	IL-6	—	0.97 (0.93–1)	0.0001	2.88	96.7 (74.4–96.2)	99 (76.4–99.1)
	CXCL-8	—	0.99 (0.9–0.9)	0.0001	99.24	96.7 (83.8–99.4)	99 (81–100.0)
	IL-10	—	0.94 (0.87–1)	0.0001	3.34	80 (63.7–90.8)	94.1 (73.0–99.0)
	TNF-α	—	0.96 (0.91–1)	0.0001	2.11	96.7 (83.8–99.4)	88.2 (65.7–96.7)

l-AUD versus ms-AUD	IL-6	—	0.77 (0.65–0.90)	0.001	0.93	71.9 (59.7–81.5)	80 (54.8–93.0)
	CXCL-8	—	0.86 (0.73–0.99)	0.0001	0.88	98.4 (91.7–99.7)	73.3 (48.0–89.1)
	TNF-α	—	0.85 (0.73–0.96)	0.0001	0.72	79.7 (67.4–88.3)	93.3 (70.2–98.8)

l-AUD versus cirrhosis	Lymphocytes	Negative	0.70 (0.59–0.81)	0.003	1.75	66.7 (53.7–75.9)	70.8 (50.8–85.1)
	Monocytes	—	0.64 (0.51–0.76)	0.046	0.48	57.6 (44.9–69.4)	62.5 (42.7–79.5)
	CD4+	—	0.72 (0.60–0.84)	0.001	36.3	69.3 (58.7–78.9)	70.4 (51.5–84.1)
	IL-6	—	0.84 (0.76–0.93)	0.0001	1.23	71.9 (59.7–81.5)	86.7 (62.1–96.3)
	CXCL-8	—	0.92 (0.84–1)	0.0001	3.55	90.6 (80.1–95.9)	80 (54.8–93.0)
	IL-10	—	0.68 (0.55–0.80)	0.034	1.12	42.2 (30.2–55.2)	93.3 (70.2–98.8)
	TNF-α	—	0.78 (0.66–0.89)	0.001	0.71	64.1 (51.2–75.2)	93.3 (70.2–98.8)

l-AUD versus AH	Monocytes	—	0.77 (0.57–0.96)	0.012	0.45	81.8 (55.2–95.3)	62.5 (42.7–79.5)
	Neutrophils	—	0.89 (0.78–1)	0.0001	4.85	81.8 (60.1–96.0)	62.5 (42.7–78.8)
	NK	Negative	0.73 (0.55–0.92)	0.022	2.35	66.7 (30.0–90.3)	88.9 (57.9–89.0)
	NKT	Negative	0.79 (0.65–0.95)	0.003	1.02	83.3 (43.6–97.0)	66.7 (62.1–91.5)
	CD4+	—	0.80 (0.65–0.95)	0.003	36.2	83.3 (49.7–91.8)	70.4 (49.8–85.4)
	IL-6	—	0.98 (0.94–0.9)	0.0001	2.5	96.7 (83.8–99.4)	99 (78–100.0)
	CXCL-8	—	0.99 (0.9–0.9)	0.0001	99.24	96.7 (83.8–99.4)	99 (77–100.0)
	IL-10	—	0.95 (0.89–1)	0.0001	2.1	93.3 (78.6–98.2)	93.3 (70.2–98.8)
	TNF-α	—	0.98 (0.93–1)	0.0001	1.85	96.7 (83.8–99.4)	93.3 (70.2–98.8)

ms-AUD versus cirrhosis	Lymphocytes	Negative	0.72 (0.64–0.82)	0.0001	1.65	63.6 (50.7–73.2)	75.3 (64.5–84.4)
	Neutrophils	Negative	0.61 (0.51–0.71)	0.025	3.85	63.6 (51.4–73.7)	63 (46.1–68.2)
	NKT	Negative	0.60 (0.51–0.69)	0.03	1.85	66.7 (55.1–76.5)	50.7 (42.7–65.5)
	CD8+	Negative	0.74 (0.65–0.82)	0.0001	20.05	81.3 (70.0–87.7)	62 (51.5–73.2)
	CD4+	—	0.65 (0.55–0.74)	0.002	37	68 (58.6–79.0)	62 (39.1–62.2)
	IL-6	—	0.67 (0.57–0.76)	0.001	1.4	68.8 (56.3–79.0)	54.7 (42.3–66.6)
	CXCL-8	—	0.72 (0.63–0.81)	0.0001	8.03	79.7 (66.1–86.7)	60.9 (50.1–73.6)

ms-AUD versus AH	Monocytes	—	0.74 (0.55–0.94)	0.009	0.59	63.6 (39.1–86.2)	78.1 (58.1–90.3)
	Neutrophils	—	0.92 (0.84–1)	0.0001	4.85	81.8 (57– 96)	71.2 (42–79)
	NK	Negative	0.81 (0.66–0.96)	0.001	8.95	75 (42.3–87.3)	77.5 (63.2–82.5)
	NKT	Negative	0.82 (0.69–0.93)	0.001	0.87	83.3 (49.2–92.2	77.5 (67.5–86.5)
	CD4+	—	0.75 (0.60–0.90)	0.005	45.6	66.7 (31.6–86.1)	85.9 (76.0–93.2)
	IL-6	—	0.94 (0.87–1)	0.0001	3.02	96.7 (83.8–99.4)	81.2 (69.1–89.2)
	CXCL-8	—	0.97 (0.93–1)	0.0001	90.44	96.7 (83.8–99.4)	93.8 (84.6–97.7)
	IL-10	—	0.84 (0.76–0.93)	0.0001	3.07	93.3 (78.6–98.2)	75 (62.4–84.5)
	TNF-α	—	0.92 (0.85–0.99)	0.0001	2.81	96.7 (83.8–99.4)	84.4 (72.7–91.6)

Cirrhosis versus AH	Neutrophils	—	0.89 (0.81–0.97)	0.0001	4.85	81.8 (60.1–96.0)	71.2 (58.9–80.1)
	NK	Negative	0.81 (0.65–0.96)	0.001	9	75 (39.8–87.3)	66.7 (55.4–77.1)
	NKT	Negative	0.79 (0.64–0.93)	0.002	0.87	83.3 (49.7–91.8)	72 (61.4–81.2)
	IL-6	—	0.79 (0.70–0.88)	0.0001	5.15	90 (74.2–96.7)	60.9 (48.6–72.1)
	CXCL-8	—	0.95 (0.91–0.99)	0.0001	90.99	96.7 (83.8–99.4)	84.4 (72.7–91.6)
	IL-10	—	0.83 (0.75–0.93)	0.0001	3.075	93.3 (78.6–98.2)	79.7 (67.3–88.3)
	TNF-α	—	0.81 (0.71–0.91)	0.0001	2.815	96.7 (83.8–99.4)	81.2 (69.1–89.2)

*Note:* Alcohol consumption groups: AUC, area under the curve of the receiver operating characteristic (ROC) curve; 95% CIs, confidence intervals. Cutoff is related to the units mentioned for each parameter.

Abbreviations: AH, alcoholic hepatitis; HD, hazardous drinking; l-AUDs, low alcohol use disorders; ms-AUDs, moderate-severe alcohol use disorders; SE, sensitivity; SP, specificity.

## Data Availability

The data that support the findings of this study are available from the corresponding author upon reasonable request.

## Supporting Information

Supporting Information 1 Figure S1. Distribution of the different study groups. Five different groups were identified, according to alcohol intake (hazardous drinking [HD], low alcohol use disorders [l-AUDs], moderate and severe alcohol use disorders [ms-AUDs], cirrhosis, and alcoholic hepatitis [AH]). The control (CT) subpopulation was adjusted to the number of alcohol-consuming subjects, in accordance with their patterns of alcohol intake. Pairwise comparisons between the alcohol consumption groups and their CT groups were performed, considering the age range as the parameter. The cirrhosis group showed statistical differences with their corresponding adjusted CT subgroup.

Supporting Information 2 Table S1. Comparison of control subjects used for each alcohol intake.

Supporting Information 3 Table S2. Peripheral blood cells and lymphocyte subpopulations in control subgroups.

Supporting Information 4 Figure S2. Representative sample of the control subjects to evaluate the lymphocyte subpopulations. (a) Discrimination of lymphocytes T and lymphocytes B. (b) Gates to analyze CD4^+^ and CD8^+^ and double positive cells (CD4^+^ and CD8^+^). (c) Selected gates to discriminate NK^+^ and NKT^+^ cells.

Supporting Information 5 Table S3. Serum concentration of cytokines in alcohol intake groups.

Supporting Information 6 Table S4. Cohen's effect size values for cytokines in alcohol intake groups.
