# Supplementary material for: Imbalance of the Immune Response According to Alcohol Consumption Patterns
Source: Mediators Inflamm. 2025 Oct 16;2025:1693583. doi: 10.1155/mi/1693583 (PMC12952229; doi:10.1155/mi/1693583)
Supplement: Supporting Information 1 — Figure S1. Distribution of the different study groups. Five different groups were identified, according to alcohol intake (hazardous drinking [HD], low alcohol use disorders [l-AUDs], moderate and severe alcohol use disorders [ms-AUDs], cirrhosis, and alcoholic hepatitis [AH]). The control (CT) subpopulation was adjusted to the number of alcohol-consuming subjects, in accordance with their patterns of alcohol intake. Pairwise comparisons between the alcohol consumption groups and their CT groups were performed, considering the age range as the parameter. The cirrhosis group showed statistical differences with their corresponding adjusted CT subgroup. [file 1693583.f1.pptx]

## Slide 1
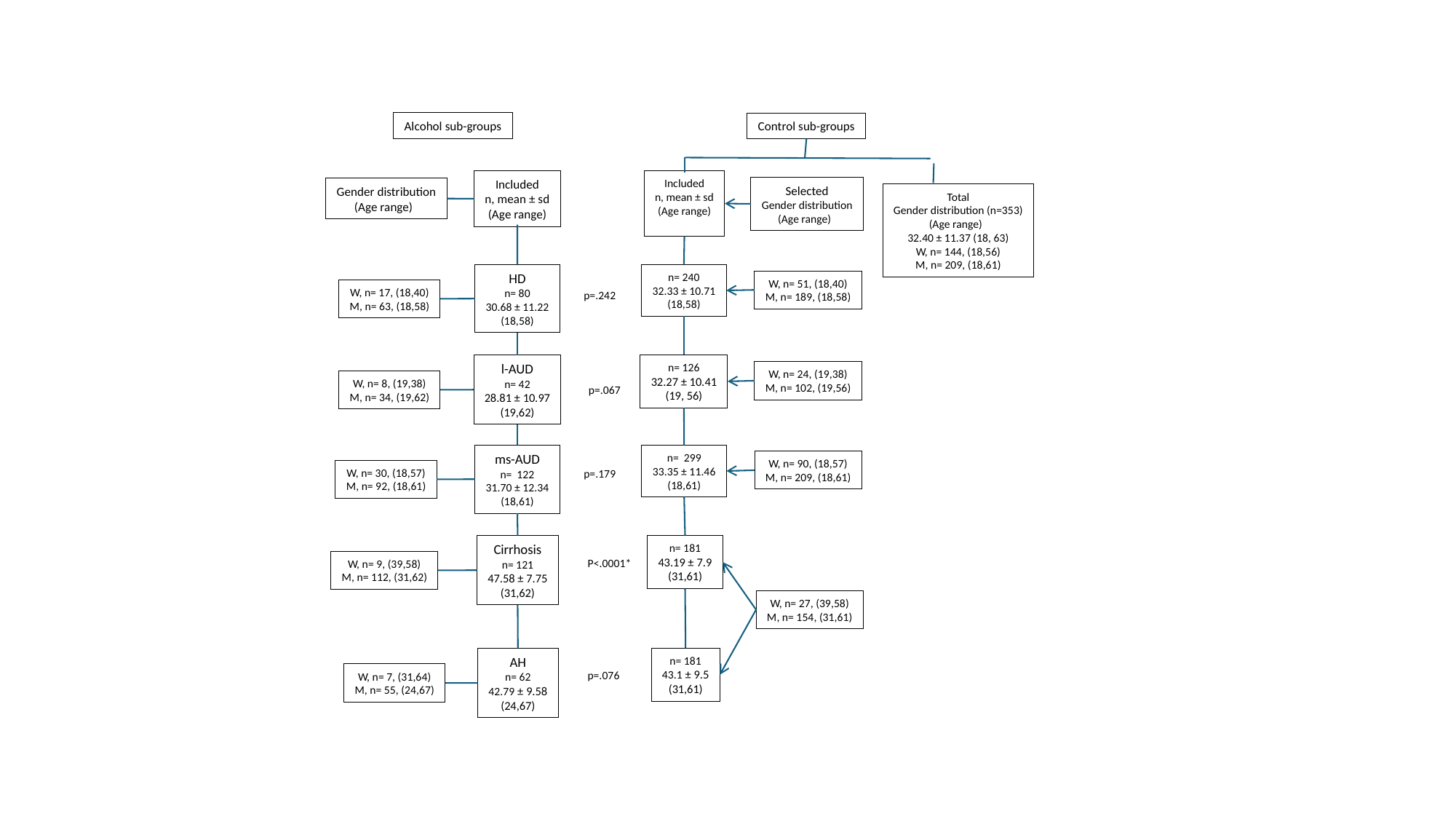

Alcohol sub-groups
Control sub-groups
Included
n, mean ± sd
(Age range)
Included
n, mean ± sd
(Age range)
Selected
Gender distribution
(Age range)
Gender distribution
(Age range)
Total
Gender distribution (n=353)
(Age range)
32.40 ± 11.37 (18, 63)
W, n= 144, (18,56)
M, n= 209, (18,61)
n= 240
32.33 ± 10.71
(18,58)
HD
n= 80
30.68 ± 11.22
(18,58)
W, n= 51, (18,40)
M, n= 189, (18,58)
W, n= 17, (18,40)
M, n= 63, (18,58)
p=.242
n= 126
32.27 ± 10.41
(19, 56)
l-AUD
n= 42
28.81 ± 10.97
(19,62)
W, n= 24, (19,38)
M, n= 102, (19,56)
W, n= 8, (19,38)
M, n= 34, (19,62)
p=.067
n= 299
33.35 ± 11.46
(18,61)
ms-AUD
n= 122
31.70 ± 12.34
(18,61)
W, n= 90, (18,57)
M, n= 209, (18,61)
W, n= 30, (18,57)
M, n= 92, (18,61)
p=.179
Cirrhosis
n= 121
47.58 ± 7.75
(31,62)
n= 181
43.19 ± 7.9
(31,61)
P<.0001*
W, n= 9, (39,58)
M, n= 112, (31,62)
W, n= 27, (39,58)
M, n= 154, (31,61)
AH
n= 62
42.79 ± 9.58
(24,67)
n= 181
43.1 ± 9.5
(31,61)
p=.076
W, n= 7, (31,64)
M, n= 55, (24,67)
