# Supplementary material for: Imbalance of the Immune Response According to Alcohol Consumption Patterns
Source: Mediators Inflamm. 2025 Oct 16;2025:1693583. doi: 10.1155/mi/1693583 (PMC12952229; doi:10.1155/mi/1693583)
Supplement: Supporting Information 2 — Table S1. Comparison of control subjects used for each alcohol intake. [file 1693583.f2.docx]

**Supplementary Table 1**. Comparison of control subjects used for each alcohol intake.

|  | **CT for HD** | **CT for**  **l-AUD** | **CT for**  **ms-AUD** | **CT for**  **cirrhosis** | **CT for AH** |
| --- | --- | --- | --- | --- | --- |
| **Age, years**  **(min, max)**  **[Q1-Q3]** | 27  (18, 58)  [22-39] | 23  (19, 31)  [27-21] | 27.5  (18, 61)  [22-39] | 42.5  (31, 61)  [37.3-49] | 38  (24, 61)  [31-45] |
| **BMI** | 25.4  (16.8, 38.9)  [23.1-28.5] | 23.8  (16.8, 31.4)  [22.2-26.7] | 25.3  (16.8, 38.9)  [23-28.4] | 27.7  (18.8, 38.9)  [25.4-30.6] | 27  (18.8, 38.9)  [24.8-29.8] |
| **AUDIT** | 2  (0, 7)  [1-4] | 3  (0, 7)  [1-5] | 2  (0, 7)  [0-4] | 2  (0, 7)  [0-4] | 2  (0, 7)  [0-4] |
| **Alcohol intake (g/occasion)** | 39  (0, 284)  [14-56.8] | 28.4  (0, 320)  [14-70] | 31.7  (0, 320)  [14-56.8] | 40  (0, 284)  [14.2-70.3] | 39  (0, 284)  [14.2-71] |
| **Alcohol intake (g/day)** | 0.9  (0, 40.6)  [0.2-2.8] | 1.9  (0, 10.1)  [0.5-4] | 0.9  (0, 40.6)  [0.1-2.8] | 0.6  (0, 40.6)  [0.1-1.9] | 0.9  (0, 40.6)  [0.1-2.4] |
| **Total Bilirubin**  **(mg/dL)** | 0.7  (0.3, 2.6)  [0.6-0.9] | 0.7  (0.3, 1.7)  [0.6-0.8] | 0.7  (0.3, 2.6)  [0.6-0.9] | 0.7  (0.3, 2.6)  [0.6-0.9] | 0.7  (0.3, 2.6)  [0.5-0.9] |
| **Direct Bilirubin**  **(mg/dL)** | 0.1  (0.1, 0.3)  [0.1-0.1] | 0.1  (0.1, 0.3)  [0.1-0.1] | 0.1  (0.1, 0.3)  [0.1-0.1] | 0.1  (0.1, 0.3)  [0.1-0.1] | 0.1  (0.1, 0.3)  [0.1-0.1] |
| **Albumin (mg/dl)** | 4.4  (3.6, 5.6)  [4.2-4.6] | 4.7  (4.1, 5.5)  [4.5-4.9] | 4.4  (3.6, 5.6)  [4.2-4.6] | 4.3  (3.6, 5.3)  [4.2-4.5] | 4.4  (3.6, 5.1)  [4.2-4.6] |
| **Glucose**  **(mg/dl)** | 93.3  (48, 237.9)  [84.8-101.5] | 88  (50, 123)  [77.5-97.4] | 92  (44.8, 237.9)  [83.5-101.2] | 96  (55.9, 237.9)  [85.7-105.8] | 94.2  (61.5, 237.9)  [85.1-103.2] |
| **AST (IU/L)** | 26.7  (12, 54)  [22-32.1] | 25  (12, 56)  [21.9-33] | 26  (12, 56)  [22-32.6] | 27  (16, 54)  [22.9-33] | 25.9  (12, 54)  [21.7-32.1] |
| **ALT (IU/L)** | 23  (7, 70)  [17-33] | 21  (7, 67)  [16.5-28] | 22  (6, 70)  [17-31] | 22  (6, 70)  [17-34] | 21  (6, 70)  [17-34] |
| **GGT (IU/L)** | 22  (9, 96)  [17-31] | 21  (9, 63)  [16-26] | 22  (5, 96)  [17-30] | 27  (7, 96)  [21-40] | 25  (7, 96)  [19-35] |

BMI, body mass index; AST, aspartate aminotransferase; ALT, alanine aminotransferase; GGT, gamma-glutamyl transferase. Control (CT), Hazardous drinking (HD), low alcohol use disorders (l-AUD), moderate and severe alcohol use disorders (ms-AUD), Alcohol hepatitis (AH). Data was expressed as median, minimum and maximum (min, max) and Q1 and interquartile ranges [Q1-Q3]. No statistical differences were identified
