# Supplementary material for: Imbalance of the Immune Response According to Alcohol Consumption Patterns
Source: Mediators Inflamm. 2025 Oct 16;2025:1693583. doi: 10.1155/mi/1693583 (PMC12952229; doi:10.1155/mi/1693583)
Supplement: Supporting Information 3 — Table S2. Peripheral blood cells and lymphocyte subpopulations in control subgroups. [file 1693583.f3.docx]

**Supplementary Table 2. Peripheral blood cells and lymphocyte subpopulations in control subgroups.**

| **A)** | **CT for HD** | **CT for**  **l-AUD** | **CT for**  **ms-AUD** | **CT for**  **cirrhosis** | **CT for AH** |  |
| --- | --- | --- | --- | --- | --- | --- |
| **Leucocytes** | 6.6  (3.4, 11.5)  [5.7-7.5] | 6.8  (4, 11.5)  [5.9-7.8] | 6.7  (2.7, 14.3)  [5.7-7.6] | 6.8  (4.1, 11)  [5.7-7.8] | 6.7  (4, 11)  [5.7-7.6] |  |
| **Lymphocytes** | 2.1  (1, 3.8)  [1.8-2.6] | 2.1  (1, 3.5)  [1.8-2.7] | 2.2  (1, 3.9)  [1.8-2.6] | 1.2  (1.2, 2.2)  [1.2-2.2] | 2.1  (1, 3.5)  [1.5-2.4] |  |
| **Monocytes** | 0.4  (0.1, 0.9)  [0.3-0.5] | 0.4  (0.1, 0.9)  [0.3-0.5] | 0.4  (0.1, 0.8)  [0.3-0.5] | 0.3  (0.2, 0.4)  [0.2-0.9] | 0.4  (0.1, 0.7)  [0.3-0.4] |  |
| **Neutrophils** | 3.6  (1.6, 8.1)  [2.9-4.4] | 3.7  (1.3, 8.1)  [3.4-4.6] | 3.6  (1.1, 7.4)  [2.7-4.4] | 3.5  (1.7, 5.4)  [1.3-4.6] | 3.2  (1.3, 7.4)  [2.6-4] |  |
| **B)** | | | | | | |
| **Flow cytometric analyses** | | | | | | |
| **CD3+** | 66.2  (39.5, 80.1)  [60.9-70.8] | 66.3  (39.5, 80)  [61.2-71.7] | 66.4  (39.5, 80.3)  [60.7-71.7] | 66.5  (47.1, 80.3)  [61.1-70.7] | 65.8  (47.1, 80.3)  [60.8-70.3] |  |
| **CD4+** | 39.6  (14.9, 59.3)  [33.7-45.8] | 38.1  (14.9, 52.4)  [33.1-42.8] | 39.3  (14.9, 59.3)  [34.5-44.8] | 42.5  (26.6, 66.6)  [37.1-47.7] | 40.9  (22.1, 66.6)  [35.3-45.4] |  |
| **CD8+** | 21.2  (9.5, 41.8)  [17.2-26.6] | 23.4  (12.5, 39)  [17.7-28.8] | 21.1  (7.1, 41.8)  [16.8-27.1] | 19.2  (7.1, 36.8)  [15.8-24.7] | 20.6  (9.5, 39)  [16.1-26.5] |  |
| **CD4+/CD8+ ratio** | 1.9  (0.4, 5.1)  [1.3-2.6] | 1.6  (0.4, 4.2)  [1.3-2.5] | 1.9  (0.4, 5.1)  [1.4-2.5] | 2.2  (0.9, 5.1)  [1.7-2.8] | 2.1  (0.6, 4.6)  [1.4-2.6] |  |
| **NK**  **(CD3-, CD16+, CD56+)** | 11.5  (1.5, 30.6)  [8.7-17.2] | 12.9  (2.9, 30.6)  [9.6-17.2] | 11.5  (1.5, 30.6)  [8.4-16.8] | 11.1  (1.5, 29.4)  [7.8-15.2] | 11.6  (1.5, 30.6)  [8-16.2] |  |
| **NKT**  **(CD3+, CD16+, CD56+)** | 1.7  (0.1, 7.4)  [1-2.7] | 1.6  (0.1, 5.8)  [0.9-2.6] | 1.7  (0.1, 7.4)  [1.1-2.7] | 1.5  (0, 7.4)  [0.9-2.4] | 1.6  (0, 7.4)  [0.9-2.7] |  |

Control (CT), Hazardous drinking (HD), low alcohol use disorders (l-AUD), moderate and severe alcohol use disorders (ms-AUD), alcohol hepatitis (AH). Leucocytes, lymphocytes, monocytes, and Neutrophils correspond to 1x10^3^/mm^3^, whereas the data for flow cytometric analyses is expressed as percentage of cells. Data was expressed as median, minimum and maximum (min, max) and Q1 and interquartile ranges [Q1-Q3]**.** No statistical differences were identified.
