# Supplementary figures and images for: Imbalance of the Immune Response According to Alcohol Consumption Patterns
Source: Mediators Inflamm. 2025 Oct 16;2025:1693583. doi: 10.1155/mi/1693583 (PMC12952229; doi:10.1155/mi/1693583)

## Slide 1
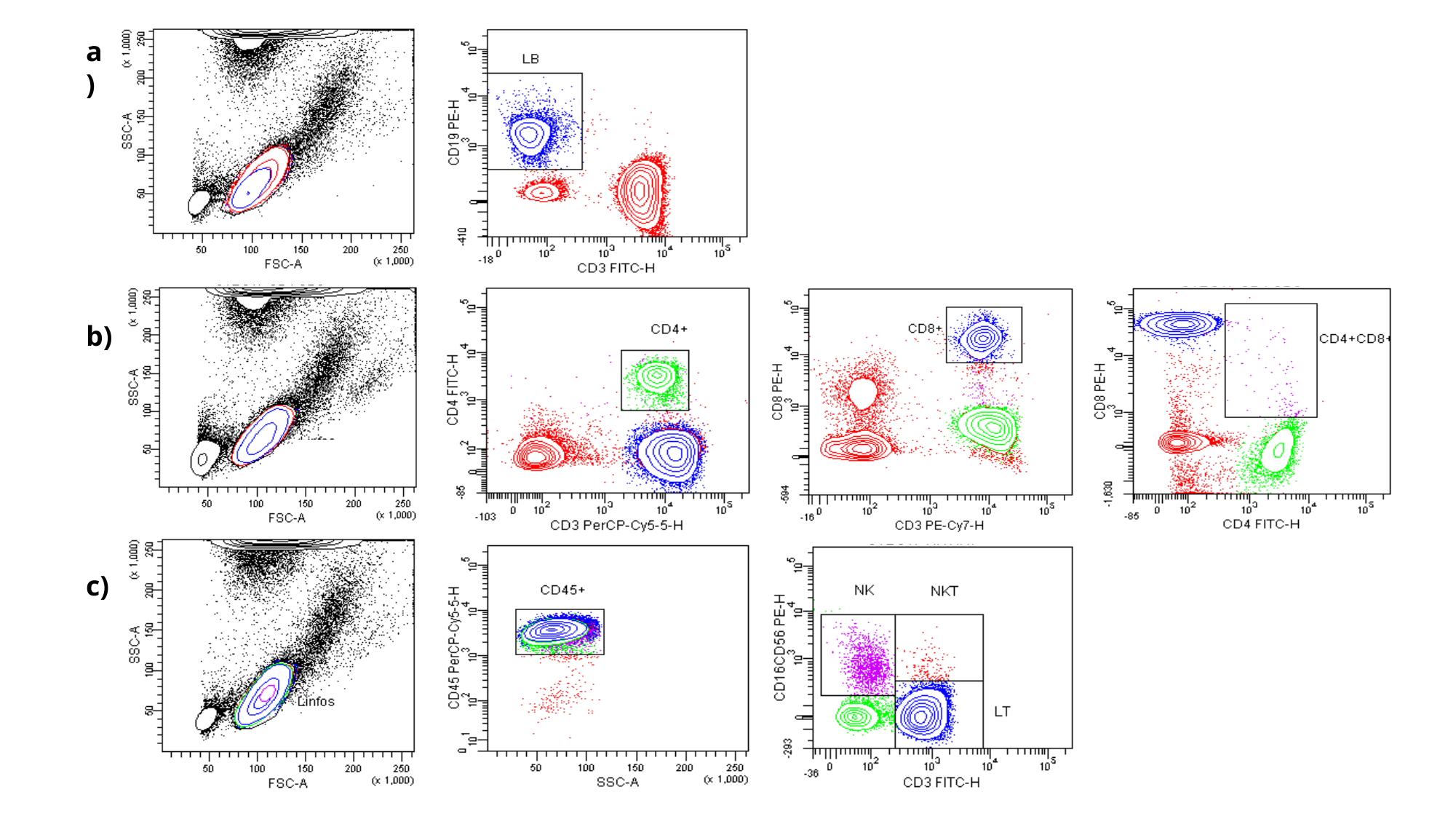

a)
b)
c)

Supplement: Supporting Information 4 — Figure S2. Representative sample of the control subjects to evaluate the lymphocyte subpopulations. (a) Discrimination of lymphocytes T and lymphocytes B. (b) Gates to analyze CD4+ and CD8+ and double positive cells (CD4+ and CD8+). (c) Selected gates to discriminate NK+ and NKT+ cells. [file 1693583.f4.pptx]
