# Supplementary material for: Imbalance of the Immune Response According to Alcohol Consumption Patterns
Source: Mediators Inflamm. 2025 Oct 16;2025:1693583. doi: 10.1155/mi/1693583 (PMC12952229; doi:10.1155/mi/1693583)
Supplement: Supporting Information 5 — Table S3. Serum concentration of cytokines in alcohol intake groups. [file 1693583.f5.docx]

**Supplementary Table 3. Serum concentration of cytokines in alcohol intake groups.**

|  | **HD** | **l-AUD** | **ms-AUD** | **Cirrhosis** | **AH** | **P value** |
| --- | --- | --- | --- | --- | --- | --- |
| **IL-2 (pg/ml)** | **2.59 ± 1.1**  (1, 21.8) | **2.32 ± 0.5**  (1, 8) | **3.48 ± 0.6**  (1, 28.6) | **3.01 ± 0.7**  (1, 26.8) | **26.5 ± 5.7**  (11.25, 65.9) | **b^ȣ^,c^*^,d^ȣ^,e^§^,f^*^,i^§^,j^*^,l^§^,n^§^,o^§^** |
| **IL-4 (pg/ml)** | **4.75 ± 0.1**  (4.5, 6.5) | **4.90± 0.2**  (4.5, 7.5) | **4.79 ± 0.1**  (4.5, 8.9) | **4.56 ± 0.05**  (4.5, 8.0) | **139 ± 15.8**  (4.5, 315) | **a^§^,e^§^,g^ȣ^,h^*^,j^ȣ^,k^*^,n^§^,o^§^** |
| **IL-6 (pg/ml)** | **1.26 ± 0.2**  (0.9, 4.7) | **1.03± 0.07**  (0.9, 1.9) | **2.72 ± 0.5**  (0.9, 32.6) | **8.7 ± 1.5**  (0.9, 57.4) | **25.7 ± 4.6**  (0.9, 118) | **a^ȣ^,c^§^,d^§^,e^§^,g^ȣ^,h^§^,i^*^,j^§^,k^§^,l^ȣ^,m^§^** |
| **IL-8/CXCL8**  **(pg/ml)** | **3.61 ± 0.5**  (0.5, 7.6) | **4.86 ± 1.2**  (0.6, 13.1) | **17** **± 3.9**  (0.8, 227) | **89.7 ± 15.8**  (3.1, 460) | **498 ± 68.8**  (43.1, 1750) | **a^*^,c^§^,d^§^,e^§^,f^ȣ^,g^§^,h^§^,i^§^,j^§^,k^§^,l^§^,m^§^,n^§^,o^§^** |
| **IL-10 (pg/ml)** | **1.6 ± 0.2**  (1.1, 3.7) | **1.3 ± 0.2**  (1.1, 3.6) | **4.9 ± 1.2**  (1.1, 48.3) | **6.7** **± 1.6**  (1.1, 71.3) | **11.2 ± 3.1**  (1.1, 59.53) | **c^ȣ^,d^§^,e^§^,i^*^,k^ȣ^,l^ȣ^** |
| **TNF-α (pg/ml)** | **1.3 ± 0.3**  (0.7, 4.8) | **2.1 ± 0.8**  (0.7, 9,2) | **3 ± 0.57**  (0.7, 30.4) | **104 ± 27.8**  (0.7, 916.7) | **22.07 ± 2.8**  (0.7, 97.5) | **b^*^,c^§^,d^§^,e^§^,f^*^,h^*^,i^§^,j^§^,k^§^,l^§^,n^§^,o^*^** |

a, CT vs HD; b, CT vs **l-AUD**; c, CT vs **ms-AUD**; d, CT vs Cirrhosis; e, CT vs AH; f, HD vs **l-AUD**; g, HD vs **ms-AUD**; h, HD vs Cirrhosis; i, HD vs AH; j, **l-AUD** vs **ms-AUD**; k, **l-AUD** vs Cirrhosis; l, **l-AUD** vs AH; m, **ms-AUD** vs Cirrhosis; n, **ms-AUD** vs AH; o, Cirrhosis vs AH. * p<0.05, significant; ^ȣ^ p<0.01. Very significant; ^§^p<0.001. Highly significant. Data was expressed as mean ± SD and minimum and maximum
