# Supplementary material for: Imbalance of the Immune Response According to Alcohol Consumption Patterns
Source: Mediators Inflamm. 2025 Oct 16;2025:1693583. doi: 10.1155/mi/1693583 (PMC12952229; doi:10.1155/mi/1693583)
Supplement: Supporting Information 6 — Table S4. Cohen's effect size values for cytokines in alcohol intake groups. [file 1693583.f6.docx]

**Supplementary Table 4.** Cohen’s effect sizes and p-values for cytokines in alcohol intake groups.

|  | | **IL2** | | **IL4** | | **IL6** | | **CXCL8** | | **IL10** | | **TNF-α** | |
| --- | --- | --- | --- | --- | --- | --- | --- | --- | --- | --- | --- | --- | --- |
| **Comparison groups** | | r | p value | r | p value | r | p value | r | p value | r | p value | r | p value |
| CT | HD | 0.05 | ns | 0.31 | 0.001 | 0.17 | 0.010 | 0.15 | 0.050 | 0.02 | ns | 0.09 | ns |
| CT | l-AUD | 0.42 | 0.010 | 0.37 | ns | 0.11 | ns | 0.26 | ns | 0.11 | ns | 0.28 | 0.050 |
| CT | ms-AUD | 0.07 | 0.050 | 0.06 | ns | 0.53 | 0.001 | 0.64 | 0.001 | 0.19 | 0.010 | 0.22 | 0.001 |
| CT | Cirrhosis | 0.18 | 0.010 | 0.13 | ns | 0.69 | 0.001 | 0.83 | 0.001 | 0.27 | 0.001 | 0.3 | 0.001 |
| CT | AH | 0.46 | 0.001 | 0.86 | 0.001 | 0.8 | 0.001 | 0.79 | 0.001 | 0.78 | 0.001 | 0.73 | 0.001 |
| HD | l-AUD | 0.39 | 0.050 | 0.02 | ns | 0.15 | ns | 0.45 | 0.010 | 0.27 | ns | 0.36 | 0.050 |
| HD | ms-AUD | 0.07 | ns | 0.32 | 0.010 | 0.29 | 0.010 | 0.37 | 0.001 | 0.09 | ns | 0.2 | ns |
| HD | Cirrhosis | 0.13 | ns | 0.28 | 0.050 | 0.45 | 0.001 | 0.58 | 0.001 | 0.15 | ns | 0.25 | 0.050 |
| HD | AH | 0.41 | 0.001 | 0.73 | ns | 0.7 | 0.050 | 0.82 | 0.001 | 0.74 | 0.050 | 0.75 | 0.001 |
| l-AUD | ms-AUD | 0.25 | 0.050 | 0.32 | 0.010 | 0.38 | 0.001 | 0.49 | 0.001 | 0.22 | ns | 0.48 | 0.001 |
| l-AUD | Cirrhosis | 0.17 | ns | 0.27 | 0.050 | 0.47 | 0.001 | 0.55 | 0.001 | 0.3 | 0.010 | 0.41 | 0.001 |
| l-AUD | AH | 0.29 | 0.001 | 0.69 | ns | 0.69 | 0.010 | 0.78 | 0.001 | 0.75 | 0.010 | 0.76 | 0.001 |
| ms-AUD | Cirrhosis | 0.08 | ns | 0.05 | ns | 0.3 | 0.001 | 0.42 | 0.001 | 0.06 | ns | 0.09 | ns |
| ms-AUD | AH | 0.39 | 0.001 | 0.87 | 0.001 | 0.63 | ns | 0.77 | 0.001 | 0.56 | ns | 0.71 | 0.001 |
| Cirrhosis | AH | 0.3 | 0.001 | 0.86 | 0.001 | 0.41 | ns | 0.64 | 0.001 | 0.5 | ns | 0.4 | 0.050 |

Control (CT), Hazardous drinking (HD), low alcohol use disorders (l-AUD), moderate and severe alcohol use disorders (ms-AUD), and alcohol hepatitis (AH). Effect size r was calculated as r Z/√ N from the Mann-Whitney U test, where N is the total number of observations. r was interpreted according to Cohen’s benchmark (0.10 = small, 0.30 = medium, 0.50 = large (blue))
